# Next-Generation Bio-Based Battery Separators: Current Status and Future Research Opportunities

**DOI:** 10.3390/gels12070650

**Published:** 2026-07-20

**Authors:** Tianyu Hu, Yunxiang Cui, Han Wang, Peiwen Liu, Qun Song

**Affiliations:** College of Engineering, Huazhong Agricultural University, Wuhan 430070, China; tianyuhu@webmail.hzau.edu.cn (T.H.); 15178327781@163.com (Y.C.); owh@webmail.hzau.edu.cn (H.W.); peiwenliu@mail.hzau.edu.cn (P.L.)

**Keywords:** biomass, battery separators, cellulose, chitin/chitosan, lignin, batteries, electrospinning, functional modification

## Abstract

Conventional polyolefin battery separators are limited by inherent deficiencies in thermal stability, electrolyte wettability, and environmental sustainability, which collectively hinder the advancement of high-energy-density energy storage systems. In this context, biomass macromolecular materials, including cellulose, chitin/chitosan, and lignin, have emerged as promising candidates for next-generation separators owing to their environmental benefits, exceptional hydrophilicity, and superior thermal resistance. This review systematically evaluates the molecular characteristics of these three biomass systems, alongside core gel-state processing and network-forming processes such as electrospinning, solution casting, nonwoven technology, and hydrogel-assisted film formation. It further highlights their cutting-edge applications in lithium-ion, lithium–sulfur, zinc-ion, and solid-state batteries, emphasizing their behavior as polymer gel electrolytes and gel-derived structural matrices. To overcome key challenges associated with mechanical robustness, interfacial compatibility, and network uniformity, advanced modification strategies are critically discussed, including surface chemical functionalization, multicomponent hybrid composite formulation, and rational three-dimensional structural engineering. Overall, current research evidence demonstrates that rationally designed biomass-based gel networks and membranes can effectively suppress metal dendrite growth, immobilize soluble polysulfide intermediates via supramolecular interactions, and reduce interfacial impedance in solid-state systems, thereby offering a viable pathway toward safer, more sustainable, and commercially competitive high-energy-density batteries.

## 1. Introduction

**Figure 1:**
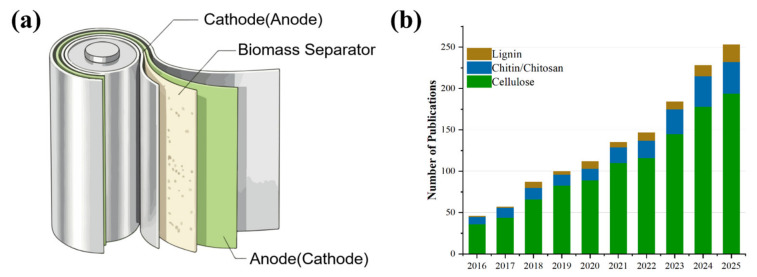
The significance and rapid development of biomass-based separators in energy storage systems. (**a**) Schematic illustration of a cylindrical battery configuration, highlighting the crucial position of the biomass separator between the cathode and anode. (**b**) The number of publications related to biomass battery separators (specifically cellulose, chitin/chitosan, and lignin) over the last decade (data collected from the Web of Science Core Collection, 2016–2025).

With the rapid development of high-energy-density and high-safety electrochemical energy storage systems, battery separators play a decisive role in overall battery performance. As key components, they isolate the anode from the cathode, prevent internal short circuits, and facilitate ion transport (Figure 1a) [1]. Currently, the commercial market is primarily dominated by polyolefin separators such as polypropylene (PP) and polyethylene (PE) [2]. However, next-generation energy storage systems demand ultimate safety, fast charging, and environmental friendliness. Against this backdrop, the inherent physicochemical limitations of polyolefin separators have become increasingly apparent. At the interfacial dynamics level, polyolefin materials consist of nonpolar hydrocarbon chains and exhibit strong hydrophobic properties. This inherent nonpolarity results in extremely poor wetting of polar electrolytes, severely hindering rapid ion transport [3]. From a thermodynamic safety perspective, polyolefin separators have an extremely low melting point. When batteries face thermal runaway or high-temperature operating conditions, the separator is prone to severe physical shrinkage or even melting, posing a high risk of catastrophic short circuits caused by direct contact between the anode and cathode [4]. Furthermore, polyolefin materials rely heavily on non-renewable petrochemical resources and lack biodegradability, which contradicts the global strategic vision of low-carbon sustainable development.

To thoroughly overcome the technical barriers of traditional polyolefin separators in terms of safety, kinetic interfaces, and ecological sustainability, the development of novel separator materials that balance high performance with sustainability has become an urgent consensus in the energy storage sector. Against this backdrop, biomass-derived hydrogels, supramolecular gels, dried gels and colloidal gels from cellulose, chitin/chitosan, and lignin are emerging as promising green raw materials for the synthesis of non-gel functional materials such as next-generation battery separators [5]. Compared to synthetic polymers, biomass fibers exhibit unparalleled intrinsic advantages. In terms of gel network topology, thanks to natural biosynthetic processes, biomass materials can naturally evolve multi-scale pore networks ranging from the micrometer to the nanometer scale, with porosity reaching over 70%, significantly surpassing traditional polyolefin separators, thereby establishing low-resistance pathways for ion transport. In terms of interfacial affinity, the surface of the biomass macromolecular framework is naturally enriched with strongly polar functional groups such as –OH and –NH_2_, endowing the separator with exceptional wettability and liquid retention capacity for polar liquid electrolytes, thereby fundamentally reducing solid–liquid interfacial resistance. In terms of thermomechanical properties, biomass macromolecules exhibit exceptional thermal stability, maintaining dimensional integrity even at temperatures far exceeding the melting point of polyolefins, thereby establishing a robust physical barrier against thermal runaway. Furthermore, as they are derived from abundant agricultural and forestry waste or marine biological resources, they significantly reduce reliance on fossil fuels, providing the material foundation for achieving a green, closed-loop life cycle in energy storage systems.

Over the past decade, research on biomass-based battery separators has experienced exponential growth, with studies centered on cellulose, chitin/chitosan, and lignin being particularly active (Figure 1b). Given the rapid evolution and interdisciplinary nature of the field, systematically reviewing its research progression and underlying scientific mechanisms holds significant theoretical significance.

While several recent reviews have provided valuable insights into biomass-derived separators, the present work offers a distinct perspective by emphasizing gel-state processing and network engineering. For instance, Xia et al. comprehensively summarized manufacturing techniques, novel biomass materials, and performance metrics across various systems [6], and Li et al. focused on bio-based engineering strategies specifically targeting metal anode stabilization [7]. We systematically evaluate how biomass macromolecules (cellulose, chitin/chitosan, and lignin) form supramolecular gels, dried gels, colloidal gels, and hydrogel-derived networks via core processes such as electrospinning, solution casting (including NIPS), nonwoven technology, and hydrogel-assisted film formation. Particular emphasis is placed on their dual roles as polymer gel electrolytes and gel-derived structural matrices that suppress metal dendrite growth through supramolecular interactions, immobilize soluble polysulfide intermediates, and reduce interfacial impedance in solid-state systems. By integrating molecular characteristics, process–microstructure–property relationships, advanced functionalization (surface modification, multicomponent hybridization into metallogels, and refined 3D structural engineering), and cross-system validation (LIBs, Li–S, Zn-ion, and solid-state batteries), this review provides new design principles and forward-looking insights into the transition from laboratory gel materials to industrially viable, high-safety, sustainable separators. This gel-network-focused framework, aligned with the scope of *Gels*, offers a complementary yet distinct contribution to the existing literature.

This review first examines the intrinsic properties of raw materials, systematically investigating the core molecular architectures of cellulose, chitin/chitosan, and lignin, and analyzing the material basis that determines the macroscopic properties of the separators. Subsequently, it provides an in-depth analysis of core fabrication processes such as electrospinning, solution casting, and nonwoven production, systematically elucidating the underlying patterns of how process conditions influence the microstructure and macroscopic properties of the separators. To address the initial mechanical or interfacial limitations of pure biomass-based membranes, this review further examines cutting-edge functionalization strategies, including surface chemical modification, multi-component hybrid composites, and refined multidimensional structural designs (e.g., gradient pores and vertical channels). After establishing the advanced material architecture, the scope is expanded to system-level application validation. The presentation will explore the superior performance of these materials in traditional lithium-ion batteries across various scenarios and provide a forward-looking assessment of their application potential and quantitative metrics in emerging energy storage systems, such as sodium/potassium-ion, lithium–sulfur, and solid-state batteries. Finally, we will critically analyze the barriers to material consistency and large-scale manufacturing currently encountered in the transition from the laboratory to industrialization, and look ahead to future trends such as smart responsive design, green continuous processing, and life cycle assessment (LCA). These biomass-derived gel networks and membranes serve as versatile platforms for constructing high-performance porous battery separators with superior thermal stability, electrolyte wettability, and dendrite suppression capability.

## 2. Sources and Core Characteristics of Biomass Fiber-Based Separators

### 2.1. Raw Material Sources and Classification

Based on resource abundance and frequency of application, biomass-based battery separator materials are primarily derived from natural polymers such as cellulose, chitin/chitosan, lignin, and a small number of other biopolymers (e.g., proteins and starch). The following provides a systematic summary of the three main biomass sources with a focus on analyzing their extraction sources, purification characteristics, and typical applications in the field of battery separators.

#### 2.1.1. Cellulose

Cellulose is widely recognized as the preferred raw material for sustainable battery separators due to its excellent mechanical strength, thermal/chemical stability, and electrolyte wettability [5]. As shown in Table 1, cellulose sources are mainly divided into three categories. Plant cellulose accounts for the vast majority of global supply. Among them, agricultural waste holds great industrial potential because of its low cost and abundant availability. However, owing to the complex lignocellulosic structure, it generally requires pretreatment prior to cellulose isolation, typically involving alkaline treatment with NaOH to remove hemicellulose and part of the lignin [8]. Subsequently, a bleaching process using H_2_O_2_ or acidified NaClO_2_ is applied to further eliminate residual lignin before subsequent fibrillation or other processing [9]. Bacterial cellulose, synthesized through microbial fermentation, features a high-purity nanofiber network, high crystallinity, and excellent mechanical properties [10]. It requires no complex pretreatment and is suitable for high-performance applications. In the future, continued advances in genetic engineering and large-scale fermentation are expected to substantially reduce production costs, enabling these materials to transition from niche, high-value additives to key components in high-performance battery applications. By contrast, cellulose derived from alternative sources such as algae, despite its inherently high crystallinity [11], remains constrained by low biomass availability and the absence of scalable cultivation and harvesting technologies, which continue to limit its industrial viability [12].

In summary, cellulose is the most practical biomass source for battery separators. Plant-derived cellulose offers the best scalability and lowest cost but requires pretreatment and exhibits only moderate mechanical strength. Bacterial cellulose provides superior purity, high crystallinity, and excellent tensile strength with uniform nanoporosity, yet its high production cost limits industrial adoption. Overall, cellulose systems deliver outstanding hydrophilicity and thermal stability, but face a clear trade-off between mechanical robustness and high ionic conductivity that requires careful structural optimization.

**Table 1:** Comparison of Major Sources of Cellulose.

Source	Representative Raw Materials	Key Characteristics	Advantages	Limitations	References
Plant Cellulose	Wood, sugarcane bagasse, Corn, crop straw, rice husks	Highest yield, containing lignin/hemicellulose	Low cost, abundant resources	Requires pretreatment	[9]
Bacterial cellulose	Microbial fermentation product	High purity, nanoscale fibers, high crystallinity	Excellent performance, no complex pretreatment	Higher production costs	[13]
Others	Algae, sea squirts	High crystallinity	High purity	Limited yield	[12]

#### 2.1.2. Chitin/Chitosan

Chitosan, a partially deacetylated derivative of chitin, possesses excellent biocompatibility, biodegradability, and a rich array of surface functional groups. Chitosan’s amino groups enable formation of ionic gels and physical gels via protonation and hydrogen bonding, which endows chitosan-based materials with unique advantages in constructing gel networks for battery separators, including tunable ion selectivity and effective dendrite suppression [14]. Furthermore, chitosan significantly improves electrolyte wettability and ionic transport kinetics, while simultaneously suppressing the polysulfide shuttle effect in lithium–sulfur batteries and promoting homogeneous lithium-ion deposition in lithium-metal anodes via electrostatic interactions. These multifaceted functionalities confer unique advantages for the development of advanced, high-safety, high-performance battery separators.

As shown in Table 2, the primary sources of chitin/chitosan include crustaceans, insects, fungi, and mollusks. Crustacean shells remain the dominant industrial source owing to their abundant availability and mature processing chains [15]. However, their high mineral and protein contents necessitate decalcification and deproteinization prior to chitin isolation [16]. Insects have attracted increasing interest as emerging sources because of their short cultivation cycles, low land requirements, and environmental benefits [17]. Nevertheless, their chitin content varies substantially with species, developmental stage, body part, and extraction protocol, complicating compositional standardization [14]. Fungal chitin offers relatively high purity, a low risk of heavy-metal contamination and allergen residues, and a stable fermentation-based supply [18]. However, its incorporation within chitin–glucan cell-wall matrices generally increases extraction and purification complexity [19]. Mollusk-derived chitin, particularly from squid pens and cuttlefish by-products, can exhibit high degrees of polymerization and favorable mechanical properties [20]. Nevertheless, its practical utilization is constrained by source-dependent availability and limited recoverable yield [21].

In summary, chitin and chitosan stand out for their amino groups, which enable unique cation selectivity and dendrite suppression. Crustacean shells remain the dominant source due to mature processing, but require decalcification and suffer from high variability in insect- and fungi-derived alternatives. While chitosan-based separators show excellent electrolyte wettability and promising performance in zinc- and sodium-ion batteries, their relatively lower mechanical strength and moisture sensitivity necessitate additional reinforcement. The key trade-off lies between valuable ion-regulation functionality and challenges in achieving consistent mechanical properties at scale.

#### 2.1.3. Lignin

Lignin’s unique three-dimensional cross-linked aromatic backbone structure gives it distinct advantages in battery separator substrate applications, effectively addressing the performance shortcomings of traditional materials and making it better suited for battery scenarios requiring high safety, high temperatures, or long cycle life. This aromatic backbone can be incorporated into hybrid gel networks or metallogels when combined with metal oxides, further expanding its functionality in advanced energy storage systems. It has a wide range of sources, primarily including black liquor from the pulp and paper industry (annual production of approximately 50–70 million tons) [22], byproducts from second-generation cellulosic ethanol production, and agricultural waste such as bagasse and crop straw. The proportions of the three primary lignin monomer units (p-hydroxyphenyl (H), guaiacyl (G), and syringyl (S)) vary significantly among different plant sources, which directly influences their reactivity, cross-linking density, and thermal stability. Specifically, softwood (coniferous) lignin is predominantly composed of G units, resulting in a highly cross-linked and rigid structure. In contrast, hardwood lignin contains both G and S units, rendering it easier to extract and richer in functional groups. Herbaceous plant lignin, however, exhibits a higher proportion of H units, leading to a looser structure that affects its purity and industrial reactivity [23].

In summary, lignin offers exceptional thermal stability and rigidity due to its aromatic backbone, making it attractive for high-safety applications. Abundant industrial waste provides a low-cost feedstock. However, pure lignin suffers from poor film-forming ability and low mechanical strength (<4 MPa). Although chemical modification and polymer blending can improve processability, these steps increase complexity and may reduce the original sustainability benefit. The main trade-off is therefore between lignin’s outstanding thermal robustness and its inherent brittleness and processing difficulties, which currently require composite strategies for practical use.

### 2.2. Intrinsic Structure and Physicochemical Properties

The intrinsic structure and physicochemical properties of biomass fibers are key factors determining their potential as substrate materials for high-safety battery separators. Cellulose, chitin/chitosan, and lignin represent three typical architectural frameworks, namely linear polysaccharides, cationic polysaccharides, and aromatic heteropolymers, respectively. These materials exhibit significant complementarity in pore formation, ion-selective transport, and thermomechanical stability. Compared to traditional polyolefin (PP/PE) separators, these biomass materials possess inherent advantages in thermal stability, hydrophilicity, and sustainability. A comparison of the intrinsic properties of the three major biomass materials and their differences from traditional polyolefin separators is shown in Table 3.

Cellulose consists of D-glucose units linked linearly via β–1,4–glycosidic bonds [29], interchain hydrogen bonding self-assembles to form semi-crystalline microfibrils [35] (CrI 40–70%) [25]. The abundant free hydroxyl groups on its surface not only provide active sites for chemical modification (such as TEMPO oxidation [36] and phosphorylation [37]) but also confer strong hydrophilicity and a tunable multi-scale pore network on the material, laying the foundation for nanoscale processing.

Chitosan, derived from deacetylated chitin, has poor solubility; its main chain contains amino groups (–NH_2_), which can form complex hydrogen-bond networks and a semi-crystalline structure [26]. Compared to cellulose with pure hydroxyl groups, the amino groups confer unique cation selectivity and dendrite inhibition capabilities to chitosan, effectively compensating for the limitations of cellulose in ion regulation [38]. In particular, after protonation in acidic electrolytes, it can effectively adsorb polysulfide ions or inhibit zinc dendrite growth.

Compared to the semi-crystalline linear chains of cellulose and the cationic structure of chitosan, the amorphous aromatic backbone of lignin confers extremely high rigidity and thermal stability on the material: the thermal decomposition onset temperature ranges from 200 to 400 °C [39], and lignin-based separators prepared by dry fibrillation exhibit no significant thermal shrinkage at 300 °C, demonstrating excellent dimensional stability [40]. This aromatic backbone structure can be incorporated into hybrid gel networks or metallogels when combined with metal oxides, offering additional pathways for interfacial regulation and performance enhancement in battery systems. Although the tensile strength of pure lignin membranes is low (<4 MPa), fiberization modification can effectively improve their brittleness [32]. Lignin’s irregular three-dimensional network structure ensures high porosity, which not only facilitates ion diffusion but also helps suppress dendrite growth [41].

In addition to cellulose, chitin/chitosan, and lignin, raw materials such as hemicellulose, starch, and proteins are also widely used in the preparation of battery separators. Hemicellulose, consisting of branched heteropolysaccharides such as xylans and mannans, features an amorphous structure and abundant hydroxyl groups, which confer strong hydrophilicity. However, its relatively low thermal stability often necessitates combination with lignin to compensate for the ionic transport limitations of cellulose or chitosan [6]. Starch has adjustable crystallinity and excellent mechanical flexibility, but its excessive hydrophilicity makes it prone to swelling. Proteins are rich in amino and carboxyl groups, providing biocompatibility and ion selectivity [38]. Algal polysaccharides contain carboxyl and sulfate groups, demonstrating gelation and dendrite suppression capabilities in aqueous zinc-ion batteries [42]. While these auxiliary biomaterials have limited film-forming capabilities on their own, they can serve as modifiers to expand performance boundaries, enhancing thermal stability, ion transport, and environmental friendliness.

### 2.3. Inherent Advantages and Limitations of Biomass Fibers as Separator Substrates

Biomass fibers possess inherent advantages such as natural gel network porosity and excellent gel hydrophilicity, making them highly promising materials for battery separators. Their highly developed micron- to nanoscale pore networks ensure electrolyte permeation and rapid ion transport without the need for complex post-processing [36]. For example, cellulose-based separators can achieve a porosity of over 70%, significantly higher than the 40–50% of polyolefin separators, effectively enhancing ionic conductivity [1]. Additionally, the surface of biomass fibers is rich in polar functional groups such as –OH and –NH_2_, which enhance affinity with polar electrolytes, reduce contact angles, promote uniform wetting, and improve ionic conductivity and cycling stability [43]. Furthermore, biomass fibers are inherently sustainable, low-cost, and environmentally friendly; their overall thermal stability is superior to that of polyolefin separators, making them less prone to thermal shrinkage at high temperatures [44], which effectively reduces the risk of thermal runaway; their excellent biocompatibility also provides a suitable foundation for flexible and biomedical batteries.

Despite these advantages, biomass fibers still suffer from several critical limitations. Their mechanical strength is generally insufficient [45]. Untreated wood pulp cellulose tends to become brittle when dry, and their tensile and puncture strengths are lower than those of polyolefin separators, making them unable to withstand assembly stresses and electrode expansion and deformation [1,46]. Natural biomass also contains impurities such as hemicellulose and pectin, necessitating complex purification to achieve battery-grade purity; otherwise, ionic contaminants can impair electrochemical stability [36]. Furthermore, biological variability in raw materials leads to inconsistent fiber diameters and crystallinity, resulting in non-uniform separator porosity and thickness that negatively affect ion transport and cell performance consistency [47]. In addition, gel swelling in electrolytes can be tuned to form stable ionic gel or organogel states and degradation in aqueous electrolytes, while the narrow electrochemical window limits applicability in high-voltage systems. Other challenges include excessive pore sizes that risk short circuits, inherent flammability, and poor film-forming properties. Therefore, there is an urgent need to address these shortcomings through process optimization and functionalization modifications to enhance the overall performance of biomass-based separators.

### 2.4. Comparison Between Biomass Separators and Commercial Polyolefin Separators

On the basis of analyzing the inherent structural merits and drawbacks of biomass substrates, we further carry out a comprehensive industrial comparison between biomass-based separators and mainstream commercial PP/PE polyolefin separators from the perspectives of production, safety, electrochemical performance and environmental sustainability.

Within lithium-ion industrial production, PP and PE polyolefin separators dominate the market by virtue of mature biaxial stretching roll-to-roll manufacturing, stable batch consistency, favorable mechanical strength and excellent chemical inertness. These separators are well compatible with automated winding and stacking assembly lines and exhibit negligible electrolyte swelling. Nevertheless, their intrinsic defects restrict their application in high-safety batteries. With melting points ranging from only 130 to 165 °C, polyolefin membranes suffer severe dimensional contraction under high temperature, which readily triggers internal short circuits and thermal runaway. Their nonpolar hydrocarbon skeletons lead to inferior electrolyte affinity, so additional ceramic coating modification is generally required. Moreover, polyolefin materials are derived from non-renewable petroleum resources and cannot biodegrade, bringing high carbon footprints and recycling costs, which contradicts the low-carbon development roadmap of the battery industry [43].

As sustainable candidates, cellulose-, chitin/chitosan- and lignin-derived separators are abundant in polar –OH and –NH_2_ functional groups. Such structural characteristics endow the membranes with outstanding electrolyte uptake and reduced solid–liquid interfacial impedance, facilitating rapid ion transport under high-rate operation. Most biomass separators maintain complete dimensional integrity above 200 °C and thus form robust physical barriers against thermal runaway. Their raw materials are renewable agroforestry and marine biowaste with biodegradable features.

Even so, multiple industrialization bottlenecks still exist. The molecular weight and fiber morphology of natural biomass fluctuate significantly with producing regions and harvesting seasons, resulting in uneven membrane thickness and disordered pore structures during continuous film formation. Lab-scale preparation methods such as electrospinning and freeze-drying suffer from low throughput and high energy consumption; wet-laid nonwoven technology requires complicated pretreatment and wastewater disposal, which greatly increases manufacturing costs. In addition, pristine biomass membranes possess insufficient tensile and puncture strength and tend to swell after long-term electrolyte immersion, which necessitates multicomponent hybridization with inorganic nanoparticles or synthetic polymers for mechanical reinforcement [48].

In summary, commercial polyolefin separators possess irreplaceable advantages in mass production and cost competitiveness at the present stage. By contrast, elaborately engineered biomass gel membranes display distinctive superiorities in thermal safety and ecological sustainability. Future research needs to break through the bottlenecks of large-scale manufacturing and long-cycle structural stability to realize partial substitution of polyolefin separators in high-safety battery scenarios.

## 3. Core Preparation Processes for Biomass Fiber Separators

### 3.1. Process Principles and Classification

The choice of preparation process for biomass fiber-based battery separators directly determines the microstructure, ionic transport efficiency, and thermodynamic stability of the separator. The three mainstream gelation and film-forming technologies currently in use—electrospinning (for nanofibrous supramolecular and organogel networks), solution casting (for physical/chemical gelation or nonsolvent-induced phase separation to form dried gels), and nonwoven technology (for colloidal gel fiber webs)—rely on core mechanisms such as nanofiber formation driven by high-voltage electric fields, precision coating via solution phase separation, and direct fiber interlacing into a web, respectively. These methods enable precise control of gel network and pore structures ranging from the nanometer to the micrometer scale for various biomass raw materials, including cellulose, chitosan, and bacterial cellulose [43].

#### 3.1.1. Electrospinning

Electrospinning is currently one of the most commonly used and structurally controllable techniques for constructing biomass nanofiber networks. Its basic principle involves extruding a solution of biomass derivatives (such as cellulose acetate or chitosan) from a syringe needle under the influence of a high-voltage DC electric field (typically 15–35 kV), forming a Taylor cone [49]. Once the electric field overcomes the surface tension of the solution, the charged jet undergoes violent flutter instability and is highly stretched and refined. As the solvent rapidly evaporates, the jet ultimately deposits on the collector to form a continuous nanofibrous supramolecular gel network [50]. These nanofibers intertwine to construct a three-dimensional, interconnected, porous network membrane.

#### 3.1.2. Solution Casting

Solution casting (also known as the doctor blade method or tape casting) is currently one of the most widely used and industrially promising solution processing technologies for the production of biomass fiber-based battery separators. The basic principle involves uniformly coating a substrate with a homogeneous solution or suspension of biomass derivatives (such as regenerated cellulose, nanocellulose, or chitosan) through a doctor blade at a precisely adjustable gap. This is followed by slow solvent evaporation (dry method) or immediate immersion into a non-solvent solidification bath to induce phase separation (wet non-solvent-induced phase separation, NIPS), thereby forming a continuous porous dried-gel film or gel membrane via phase separation or gelation [48]. In the wet NIPS process, rapid bidirectional diffusion between the solvent and the non-solvent triggers instantaneous liquid–liquid phase separation, resulting in a structure comprising a polymer-rich phase (scaffold) and interconnected polymer-poor phases (pores). This process is governed by both the Flory–Huggins thermodynamic phase diagram and diffusion kinetics, and the porosity and pore size distribution can be precisely controlled by adjusting solution concentration, doctor blade gap, and the temperature and composition of the solidification bath [51]; in the dry method, molecular chain self-assembly is achieved through shear-thinning rheological properties (low shear and high viscosity maintain shape, while high shear and low viscosity facilitate coating) [52]. These porous films typically exhibit good thickness uniformity and hydrophilicity. The solution casting process offers significant advantages, including simple equipment, precise thickness control, low cost, and ease of continuous roll-to-roll production.

#### 3.1.3. Nonwoven Technology

Nonwoven technology (also known as the nonwoven process or wet/dry web formation technology) has become one of the most economical and scalable film-forming methods for preparing biomass fiber-based battery separators. This process is primarily divided into two major stages: web formation and consolidation. Among these, wet-laid or papermaking processes have become the most mainstream method for preparing battery separators due to their high compatibility with the natural hydrophilicity of biomass cellulose. The basic principle involves dispersing biomass fibers (cellulose nanofibers or wood pulp) in an aqueous medium, forming a colloidal gel web or fiber gel network through vacuum filtration and consolidation, and then consolidating it via hydroentanglement or hot-pressing [53]. Dry-laid web formation, also known as air-laid web formation, involves conveying individual short fibers via an air stream onto a collection belt to form a loose fiber mat or web. Subsequently, the resulting fiber mat can be consolidated using mechanical, thermal, or chemical methods [54]. Dry-laid web formation is more environmentally friendly as it does not require a water medium and is suitable for the preparation of flexible composite separators; however, its application in pure biomass systems is relatively limited, and its uniformity is slightly inferior to that of the wet-laid method. With mature equipment, extremely low costs, low energy consumption, and natural compatibility with natural biomass fibers, this process directly utilizes overlapping fibers to form a web, making it particularly suitable for the rapid industrial production of natural long-fiber raw materials such as wood pulp cellulose and bacterial cellulose. It forms a striking complement to strategies such as electrospinning, which relies on high-voltage electric fields to construct nanofiber networks, and solution casting, which achieves precise coating through solution phase separation.

#### 3.1.4. Hydrogel-Assisted Film Formation and Gelation Strategies

Hydrogel-assisted film formation can serve as a complementary approach to electrospinning, cast film formation and nonwoven technologies. Its underlying principle relies on utilizing polar functional groups, including hydroxyl, amino, and carboxyl groups, along biomass polymer chains to induce a sol–gel transition. Driven by hydrogen bonding, ionic coordination, or covalent cross-linking, this transition directly constructs a continuous three-dimensional (3D) hydrated network, distinguishing it from conventional film-forming mechanisms that depend on fiber deposition or phase separation [55]. Depending on the nature of the cross-linking mechanism, hydrogel networks are primarily classified as either physically or chemically cross-linked. Physical cross-linking relies on reversible non-covalent interactions, such as hydrogen bonding, molecular chain entanglement, and ionic coordination, to form dynamic networks [56]. Within polysaccharide gel systems, ionic cross-linking represents a distinct and crucial form of physical cross-linking. It fundamentally involves the coordination of multivalent metal ions with functional groups along the polymer backbones a classic example being the “egg-box” structure formed by alginate and divalent cations [57]. Conversely, chemical cross-linking establishes permanent, highly stable networks via covalent bonding. From a process perspective, hydrogel-assisted film formation typically comprises four steps: preparation of the film-forming solution, gelation induction, film shaping and post-treatment. The resulting network topology is highly sensitive to critical parameters including polymer concentration, cross-linking density, and post-treatment protocols [58]. In essence, by synergistic coupling of the gelation-driven 3D network construction with precise post-treatment regulations, this strategy allows for flexible tailoring of wet/dry film architectures, thereby providing versatile and scalable routes for manufacturing biomass-based battery separators.

### 3.2. Influence of Process Parameters on the Microstructure and Mechanical Properties of Membranes

Precise control of process parameters is critical in determining the microstructure and macroscopic properties of the biomass fiber-based membrane matrix. Parameters such as solution concentration, voltage, and drying/solidification conditions directly regulate pore structure, pore size distribution, fiber diameter, thickness, and basic mechanical strength by influencing phase separation kinetics, fiber entanglement, or stretching behavior. These structural features not only inherit the intrinsic properties of biomass fibers (such as the –OH hydrogen-bonding network of cellulose and the cationic functional groups of chitin) but also directly influence subsequent ion transport efficiency, thermal stability, and electrochemical performance.

#### 3.2.1. Influence of Electrospinning Process Parameters

Parameters such as solution concentration, applied voltage, flow rate, take-up distance, and ambient humidity precisely control the diameter, morphology, and three-dimensional network structure of nanofibers by regulating Taylor cone stability, jet flutter, and solvent evaporation kinetics. Solution concentration is the most critical control factor: at too low a concentration, surface tension dominates, leading to the formation of bead-like defects and a significant increase in fiber diameter [59]; conversely, as concentration increases, viscosity rises and molecular chain entanglement intensifies, leading to larger fiber diameters and lower porosity, though network interconnectivity improves [60]. The applied voltage promotes jet refinement and fluttering by enhancing electrostatic forces; increasing the voltage can reduce fiber diameters to the submicron range, resulting in a more uniform pore size distribution and higher porosity [61]. Increased flow velocity tends to cause jet instability and coarsen the fibers [62], while extending the receiving distance promotes more thorough fluttering and a narrower pore size distribution. Higher humidity and temperature slow solvent evaporation, resulting in a looser network but enhanced interconnectivity (this effect is amplified by hydrophilic functional groups in the biomass) [63]. Synergistic optimization of the above parameters can significantly improve electrolyte wettability, suppress dendrite growth, and result in a thermal shrinkage rate far lower than that of commercial polyolefin separators, thereby ensuring high safety and high-rate performance.

#### 3.2.2. Influence of Parameters in the Casting Process

In the solution casting process (especially wet NIPS), factors such as solution concentration, doctor blade gap, solidification bath temperature/composition, and drying conditions determine the pore morphology, pore size distribution, and thickness uniformity of the membrane by regulating the phase separation rate and molecular chain self-assembly. As the polymer concentration in the casting solution increases, the system viscosity rises significantly, thereby slowing the solvent-nonsolvent exchange rate. This leads the phase separation process to form structurally uniform, sponge-like micropores, which not only improves the consistency of the porosity distribution but also reduces the total porosity to some extent [64]. The blade gap directly controls the final film thickness; while a larger gap facilitates the preparation of thicker films, it tends to increase thickness deviations and reduce mechanical strength [65]. An increase in the solidification bath temperature or the strength of the desolvent significantly accelerates the instantaneous phase separation process, forming finger-like macropores that significantly facilitate rapid ion transport; conversely, slow separation under low-temperature or weak desolvent conditions generates dense sponge-like pores, thereby improving tensile strength [66]. Drying/annealing further refines the pore structure by regulating crystallinity. Due to the abundant hydrophilic functional groups in biomass cellulose/chitosan, phase separation behavior is more sensitive to these parameters, ultimately enabling the production of a base film with uniform thickness and controllable pore size, combining excellent wettability with thermal shrinkage suppression.

#### 3.2.3. Influence of Nonwoven Processing Parameters

In nonwoven technologies (especially wet-laid web formation), fiber dispersion concentration, vacuum filtration pressure, and hot-pressing pressure and temperature are the core process parameters for controlling fiber network density, pore structure, and thickness. By influencing fiber interlacing density and consolidation behavior, these parameters directly determine the microstructure and macroscopic properties of the base film. A lower slurry concentration combined with higher vacuum filtration pressure can form a loose, three-dimensional interconnected network, thereby significantly improving electrolyte absorption and ionic conductivity [67]. Conversely, increasing hot-pressing pressure and temperature compresses the fiber network, significantly reducing thickness and narrowing the pore size distribution, while substantially enhancing tensile strength, achieving a good balance between strength and porosity [68]. Due to their natural long-fiber characteristics, bacterial cellulose and wood pulp raw materials respond more effectively to these parameters. Although dry-laid web formation involves similar parameters (airflow velocity, consolidation temperature), the uniformity of the network is slightly inferior. Systematic optimization of nonwoven technical parameters achieves a balance between porosity, submicron pore size, and high mechanical strength, endowing the biomass membrane with excellent hydrophilicity, thermal stability, and ionic transport capability.

#### 3.2.4. Influence of Hydrogel-Assisted Fabrication Parameters

The structural characteristics and membrane properties of hydrogel-assisted films are primarily determined by key parameters including polymer concentration, crosslinking density, gelation rate, and post-processing methods [69]. An elevated polymer concentration enhances molecular chain entanglement and film continuity. However, excessively high concentrations markedly increase system viscosity, which compromises coating uniformity and impedes ion diffusion. Moderate increases in crosslinking density improve the dimensional stability and swelling resistance of the gel network, whereas excessive crosslinking compresses swelling space and reduces pore connectivity, thereby impairing electrolyte absorption and ion transport efficiency [70]. Gelation kinetics play an equally vital role. Rapid gelation often induces localized crosslinking gradients that result in heterogeneous networks, whereas a controlled and decelerated gelation process facilitates molecular chain reorganization to yield a more homogeneous structure [71]. Additionally, pH, temperature, and ion strength significantly influence network density and stability by modulating functional group ionization states, hydrogen bond strength, and ion coordination effects. During post-processing, freeze-drying tends to preserve open-pore structures and supercritical drying effectively prevents network collapse, whereas atmospheric drying or thermal compression typically results in denser membrane structures.

### 3.3. Process Adaptation for Different Raw Materials

Biomass fibers exhibit significant differences in molecular structure, solubility, and dispersion behavior, which directly determine their compatibility with mainstream fabrication processes such as electrospinning, solution casting, and wet nonwoven processes, thereby profoundly influencing core properties of the membrane matrix, including porosity, pore size distribution, fiber diameter, mechanical strength, and thermal stability. Therefore, this section systematically analyzes the optimal matching strategies between typical raw materials (such as plant cellulose, bacterial cellulose, chitin/chitosan, and lignin) and each fabrication process. By analyzing the intrinsic relationships between process parameters, raw material properties, microstructure, and macro-performance, this section clarifies how to fully exploit the inherent advantages of each biomass material, such as the excellent water-based dispersibility of plant cellulose and the intact three-dimensional network of bacterial cellulose. The cationic polyelectrolyte properties of chitosan and the high thermal stability of aromatic lignin provides a solid process foundation for subsequent surface modification, multi-component composites, and refined structural design.

#### 3.3.1. Processes Suitable for Plant Cellulose

After pretreatment to remove lignin and hemicellulose (Table 1), plant cellulose exposes abundant polar hydroxyl groups, conferring excellent aqueous dispersibility. This characteristic renders it highly compatible with nonwoven technology. During vacuum filtration at low slurry concentrations, the abundant surface hydroxyl groups undergo hydrogen-bonded self-assembly to form a three-dimensional interconnected fiber network [72]. The resulting separator simultaneously achieves high porosity, excellent hydrophilicity, and retention of intrinsic mechanical strength and thermal stability. For example, Lv et al. [67] introduced nanocellulose as a pore-regulating agent into a micron-scale scaffold via co-filtration, precisely tuning pore distribution and raising the tensile strength of the composite separator to 49 MPa, thereby constructing a robust barrier against lithium dendrite growth. Similarly, Zhou et al. showed that vacuum-filtered cotton-based pure cellulose membranes exhibited a tensile strength of 29.2 MPa together with a perfect electrolyte contact angle of 0°. The surface-enriched polar hydroxyl groups markedly lowered the desolvation energy barrier of metal ions, homogenizing deposition flux and effectively suppressing dendrite formation [73].

As summarized in Table 4, nonwoven cellulose separators fabricated by vacuum filtration exhibit clear advantages in tensile strength (29.2–49 MPa) and electrolyte affinity over those prepared by electrospinning and NIPS. In addition, the process is entirely solvent-free, which aligns with the principles of green and sustainable manufacturing.

In contrast, direct application of solution casting and electrospinning to plant cellulose-based battery separators faces significant limitations. Both approaches generally require harsh organic solvent systems or chemical derivatization pretreatments. Zhu et al., for instance, prepared CA/PVDF composite membranes by electrospinning from a DMAc/acetone mixed solvent (3:7 *v*/*v*) [74]. Likewise, Asri et al. fabricated CA/chitin nanofiber composite membranes via solvent-induced phase inversion using a DMAc/acetone system, followed by alkali deacetylation (0.06 M NaOH) to enhance electrolyte stability [75].

**Figure 2:**
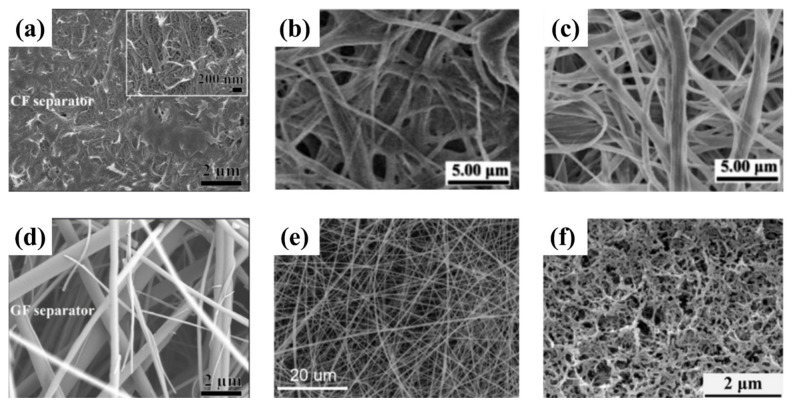
SEM morphologies of plant cellulose-based separators prepared by different methods, demonstrating the superiority of the nonwoven filtration technique. (**a**–**c**) Separators fabricated by vacuum filtration (nonwoven method) exhibit dense, uniform nanopores and well-interconnected three-dimensional fiber networks, which are beneficial for high electrolyte affinity and mechanical strength. (**a**) Cotton-derived cellulose film (CF) separator; (**b**,**c**) Cellulose nanofibril (CNF)-reinforced cellulose paper separators with tunable pore structures. (**d**) Commercial glass fiber (GF) separator showing large and irregular pores, in stark contrast to the nonwoven samples. (**e**) Electrospun CA/PVDF composite membrane with randomly oriented nanofiber network. (**f**) Nanoporous regenerated cellulose separator prepared by nonsolvent-induced phase separation (NIPS). Scale bars are indicated in each panel. Panels (**a**,**d**) are reproduced from Ref. [73] with permission from Elsevier Ltd., copyright 2022. Panels (**b**,**c**) are reproduced from Ref. [67] with permission from Elsevier Ltd., copyright 2021. Panel (**e**) is reproduced from Ref. [74] with permission from Elsevier Ltd., copyright 2024. Panel (**f**) is reproduced from Ref. [76] with permission from the American Chemical Society, copyright 2021.

Microstructural characterization in Figure 2 provides direct visual evidence for these performance differences. Vacuum-filtration-derived nonwoven cellulose separators (Figure 2a–c) display dense, uniform nanopores and well-interconnected three-dimensional fiber networks. By comparison, commercial glass-fiber separators exhibit large and irregular pores (Figure 2d), electrospun CA/PVDF membranes show randomly oriented nanofiber networks (Figure 2e), and NIPS-prepared regenerated cellulose membranes present a sponge-like morphology (Figure 2f). This superior microstructure underpins the higher tensile strength and electrolyte wettability of nonwoven separators.

**Table 4:** Comparison of the performance of cellulose-based separators prepared by different methods.

Preparation Method	Electrolyte Absorption (%)	Tensile Strength (MPa)	Porosity (%)	Ionic Conductivity (mS cm^−1^)	Contact Angle (°)	References
NIPS	436	13.4–21.6	51–61	0.90–1.25	16.1–26.1 (Water)	[76]
NIPS combined with alkali treatment	873.89	4.12–13.31	54.86	0.57	–	[75]
Electrospinning	97.1	1.6	76.9	1.82	–	[74]
Electrospinning + casting	–	13.76	–	0.104	90 (water)	[77]
Nonwoven Technology	136.4	49	–	0.71	19.52 (electrolyte)	[67]
Nonwoven Technology (Aqueous zinc electrolysis)	–	29.2	–	56.95	0(electrolyte)	[73]
Nanofibers Nonwoven technology	–	42	–	0.75	–	[72]

#### 3.3.2. Bacterial Cellulose Processing

Bacterial cellulose requires no complex pretreatment; its native three-dimensional interconnected nano-network can be formed in the wet state through simple vacuum filtration, freeze-drying combined with hot pressing, or direct hydrogel casting, without the need for solvent dissolution or additional dispersion. This process fully preserves the native high porosity, uniform submicron pore size distribution, and extremely high thermal stability and mechanical strength [78]. For example, Hu et al. directly utilized a native supramolecular hydrogel pellicle with three-dimensional cross-linked colloidal gel network through freeze-drying and hot-pressing, achieving a high porosity of 74% and zero thermal shrinkage at 200 °C, demonstrating excellent structural integrity [79]. Similarly, Chen et al. employed a simple vacuum filtration process combined with room-temperature drying to directly shape the native hydrogel. This not only fully preserved the uniform submicron-scale pore structure but also converted the bacterial cellulose’s tensile strength of up to 207 MPa into macroscopic membrane performance, enabling ultra-long-term stable cycling exceeding 5200 h in aqueous zinc-ion batteries [80].

Compared to plant cellulose, bacterial cellulose has a higher degree of crystallinity and a denser, more well-developed hydrogen-bond network. Consequently, the challenges faced by plant cellulose in electrospinning and solution casting—such as dissolution difficulties, structural damage, and performance degradation—are particularly pronounced in bacterial cellulose [81]. More importantly, the intrinsic physical morphology of bacterial cellulose determines its fundamental incompatibility with nonwoven processes used for plant cellulose. Plant cellulose requires vigorous mechanical pulping and disintegration before self-assembling into a network via hydrogen bonding in an extremely low-concentration suspension [67]; in contrast, bacterial cellulose can form a complete three-dimensional hydrogel film (pellicle) directly in the culture medium without any pulping or dispersion treatment [82]. If the plant cellulose route is forcibly adopted and the native pellicle is subjected to high-shear disruption to form a slurry, its continuous network of ultra-long nanofibers will be irreversibly destroyed [83]; even if subsequent filtration and reassembly are performed, the resulting structure relies only on weak physical entanglement rather than the native topological interlocking, leading to a significant decline in ultimate tensile strength and the connectivity of ion channels [84]. Hydrogel-assisted film formation and direct vacuum filtration are preferred for bacterial cellulose because they preserve the native three-dimensional interconnected nanofiber network without high-shear disruption, maintaining both ultra-high mechanical strength and uniform submicron porosity that would be lost in conventional slurry-based nonwoven processes.

#### 3.3.3. Chitosan Adaptation Process

The chitosan backbone is rich in –NH_2_ and –OH groups; in dilute acid solutions, its amino groups are highly prone to protonation, thereby forming a cationic polyelectrolyte that confers high charge density and suitable viscoelastic and rheological properties to the solution. This characteristic endows chitosan with excellent gel-forming ability, enabling the formation of ionic gels and physical gels in dilute acid through protonation and hydrogen bonding. Consequently, chitosan is particularly suitable for electrospinning to produce nanofibrous gel networks; simultaneously, its excellent film-forming ability and controllable phase separation properties also make it highly suitable for solution casting processes to prepare gel membranes or dried gels. For example, Hassan et al. [77] prepared a composite separator via electrospinning combined with chitosan solution casting, which exhibited a high mechanical strength of 13.76 MPa and a sodium-ion conductivity of 1.04 × 10^−4^ S cm^−1^ in flexible solid-state sodium-ion batteries; Wu et al. [85] prepared a chitosan–zinc gel electrolyte membrane using solution casting combined with Zn^2+^ coordination and mechanical compaction, which exhibited an ionic conductivity as high as 72 mS cm^−1^ and achieved excellent dendrite suppression and ultra-long cycle stability (over 1000 cycles at 50 mA cm^−2^) in high-rate aqueous zinc metal batteries.

In contrast, nonwoven technology is unsuitable for the preparation of battery separators from chitosan primarily because chitosan struggles to form a stable fiber suspension in aqueous media; it is highly prone to partial dissolution, agglomeration, or uneven dispersion. This is incompatible with the process requirements of nonwoven technology, which heavily relies on the uniform dispersion of preformed long fibers and their self-assembly into a network via hydrogen bonding. Song et al. [28] explicitly point out that, unlike natural cellulose, chitosan lacks raw fiber materials that can be directly used in large-scale papermaking processes; even when using regenerated wet-spun fibers, complex pretreatment is still required to barely form a web. Electrospinning and solution casting are favored for chitosan due to its excellent solubility in dilute acid and good film-forming ability, which allow the construction of nanofibrous or dense gel membranes with tunable cationic functionality. Nonwoven technology is generally unsuitable because chitosan lacks stable long-fiber raw materials and tends to agglomerate or partially dissolve in aqueous media.

#### 3.3.4. Lignin Adaptation Process

Lignin possesses a highly cross-linked three-dimensional aromatic backbone structure. Its molecular chains are rigid, its solubility is limited, and its intrinsic film-forming ability is extremely poor. When processed alone, it is highly prone to brittle fracture and struggles to form continuous, uniform films. This characteristic makes it difficult to form films independently using solution casting or nonwoven processes; however, electrospinning can significantly improve the rheological properties of the spinning solution by blending lignin with flexible spinning aids. For example, Song et al. [86] prepared a nanofiber separator via electrospinning of a blend of sulfate lignin and polyimide (PI), which formed a three-dimensional interconnected network with uniform pore diameters. The separator achieved an electrolyte absorption rate of 592%, an ionic conductivity as high as 1. 78 × 10^−3^ S cm^−1^ and a lithium ion transference number of 0.787, with all key performance metrics significantly outperforming commercial Celgard separators. Yerkinbekova et al. [41] further employed maleic anhydride-modified lignin blended with polyacrylonitrile (PAN) and UV-crosslinked via electrospinning. The resulting nanofiber separator achieved an electrolyte absorption rate of 1180.8% and an ionic conductivity of 2.79 × 10^−3^ S cm^−1^. It exhibited zero thermal shrinkage at 150 °C and achieved stable cycling for over 1000 h in a Li||Li symmetric cell, comprehensively outperforming commercial Celgard separators. Electrospinning is the most practical route for lignin because its rigid aromatic structure and poor inherent film-forming ability make direct solution casting or nonwoven processing extremely difficult. Blending with flexible polymers (e.g., PI or PAN) during electrospinning significantly improves spinnability while retaining lignin’s high thermal stability.

### 3.4. Comparative Evaluation and Selection Guidelines for Fabrication Routes

As summarized in Table 5, the choice of fabrication route should be guided by the target biomass material and performance priorities. Nonwoven technology offers the best balance of scalability and mechanical strength for plant cellulose, while electrospinning is preferred when nanoscale structural precision and ion selectivity are critical. Hydrogel-assisted methods are uniquely advantageous for preserving the native network of bacterial cellulose.

## 4. Functionalization and Modification Strategies for Biomass Fiber Separators

### 4.1. Surface Modification and Coating

Electrospinning, solution casting, and nonwoven technologies have successfully produced high-porosity, naturally hydrophilic, and structurally controllable biomass fiber-based membranes. However, these base membranes still suffer from notable inherent limitations. Plant cellulose nonwoven membranes, despite their excellent hydrophilicity, exhibit insufficient mechanical strength and inadequate control over thermal shrinkage. Bacterial cellulose direct-film membranes, although possessing outstanding mechanical properties, offer limited process flexibility. Chitosan-based membranes are prone to moisture absorption and generally display low mechanical strength. Lignin-based membranes suffer from poor film-forming ability and typically require composite reinforcement. Therefore, the base membranes prepared in Chapter 3 require further functionalization strategies to achieve a deep integration of intrinsic material properties with tailored process structures.

Surface modification and coating strategies offer effective pathways to address these bottlenecks. The core advantage of these strategies lies in their ability to selectively regulate surface chemical composition, thermomechanical properties, and ion transport behavior through methods such as plasma activation, chemical grafting, or thin-film deposition, without compromising the integrity of the main porous network. This approach enables the simultaneous optimization of electrolyte wettability, thermal stability, and interfacial compatibility. This subsection provides a systematic examination of the underlying mechanisms, material compatibility, and representative case studies associated with three principal surface modification strategies, namely plasma treatment, chemical grafting, and ceramic/polymer coatings. These analyses establish the theoretical and practical foundation for the subsequent development of multi-component composite systems and refined structural architectures.

#### 4.1.1. Surface Chemical Modification

The core mechanism of surface chemical modification lies in regulating solid–liquid interface behavior through the targeted conversion of functional groups, including reducing the desolvation energy barrier of cations, homogenizing ion flux to inhibit dendrite growth, chemically trapping harmful byproducts, and optimizing interfacial wettability. Due to significant differences in intrinsic functional groups and skeletal structures among various biomass feedstocks (cellulose is dominated by hydroxyl groups, chitosan contains both amino and hydroxyl groups, and lignin contains phenolic hydroxyl groups and an aromatic skeleton), distinct modification adaptation mechanisms and performance enhancement pathways emerge based on the base films prepared in Chapter 3. The following sections will systematically discuss specific strategies and recent advancements in surface chemical modification, focusing on three major categories of biomass base films: cellulose, chitin/chitosan, and lignin.

**Figure 3:**
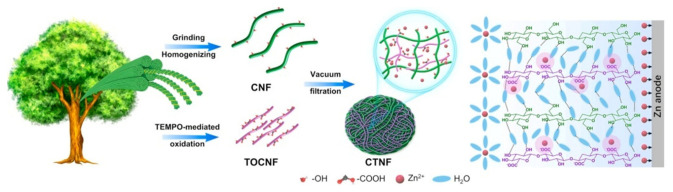
Schematic illustration of the CTNF separator preparation and the synergistic hydroxyl–carboxyl chemistry for regulating Zn^2+^ desolvation and dendrite-free deposition. The black arrows indicate the migration and subsequent deposition of Zn^2+^ toward the Zn anode. Reproduced from Ref. [87] with permission from Elsevier B.V., copyright 2025.

**Figure 4:**
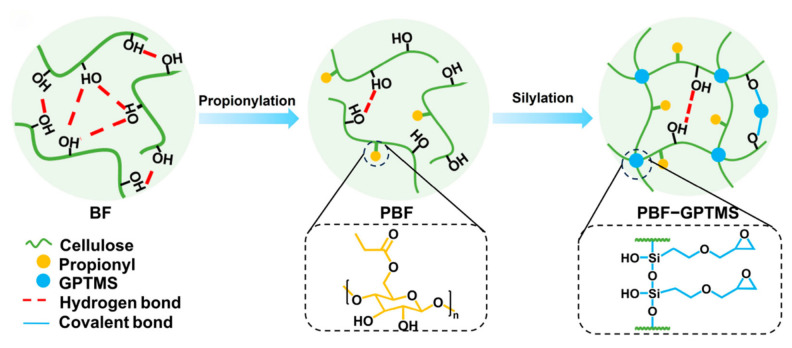
Schematic of the fabrication process for the silane-crosslinked PBF-GPTMS cellulose separator, highlighting enhanced wet strength and suppressed HF formation. Reproduced from Ref. [88] according to the Creative Commons Attribution 4.0 International License.

The surface of linear β–1,4–glucan chains in cellulose is rich in hydroxyl groups, providing abundant active sites for functional group oxidation and chemical grafting. Introducing carboxyl groups via TEMPO-mediated oxidation or performing graft modification using silane coupling agents allows for the regulation of interfacial chemical composition at the molecular level, thereby optimizing ion transport properties and interfacial stability. As illustrated in Figure 3, Wang et al. [87] employed a TEMPO-mediated oxidation strategy to selectively convert cellulose C6 hydroxyl groups into carboxyl groups, constructing a hydroxyl–carboxyl synergistic network that significantly enhanced zinc ion selectivity. The Zn^2+^ with a migration number of up to 0. 70, and the symmetric Zn||Zn battery achieved a stable cycle life exceeding 6400 h, effectively suppressing zinc dendrite growth. Turossi et al. [29] extended the TEMPO oxidation strategy to the modification of cellulose nanocrystals; the resulting separator exhibited nearly zero thermal shrinkage at 200 °C, effectively addressing the shortcomings in thermal safety performance of traditional cellulose separators. Furthermore, Cui et al. [88] employed GPTMS as a silane coupling agent to graft and crosslink propylated cellulose fibers. As illustrated in Figure 4, the resulting Si–O–C network significantly improves wet strength while effectively capturing HF generated from LiPF_6_ hydrolysis. This dual benefit reduces side reactions at the anode interface and enables excellent long-term cycling stability in lithium metal batteries. These surface chemical modifications play a critical role in regulating the supramolecular gel network stability and ionic gel properties upon electrolyte swelling.

**Figure 5:**
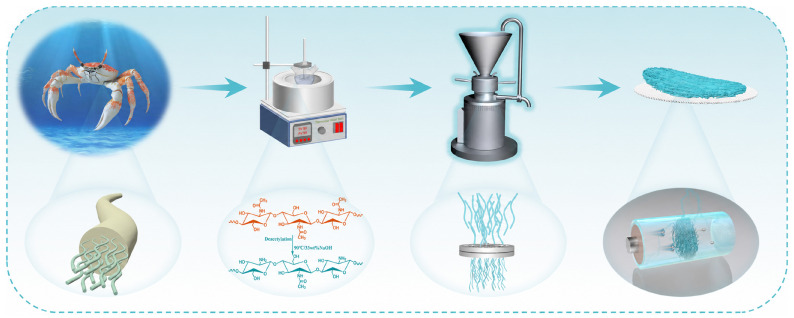
Schematic diagram of the preparation of D-x-ChNF separators via alkaline deacetylation and mechanical grinding, showing enhanced Zn^2+^ coordination with increasing amino group content. Reproduced from Ref. [89] according to the Creative Commons Attribution—NonCommercial-NoDerivatives 4.0 International License, copyright 2025, published by Springer Nature.

Unlike cellulose, which has a purely hydroxyl-based structure, chitin inherently possesses both hydroxyl and amino active sites. By leveraging the charge-modulation advantages of nitrogen-containing functional groups, deacetylation and oxidative grafting modifications can achieve multiple improvements in ion selectivity, uniform deposition, and cycling stability. As shown in Figure 5, Lin et al. [89] induced partial deacetylation of chitin via alkali treatment, enriching the fiber surface with highly reactive amino groups. The protonation of amino groups is proposed to reduce the zinc nucleation overpotential, which may contribute to more uniform Zn^2+^ deposition and the observed extension of cycle life in symmetric cells. TEMPO oxidation has also been applied to chitosan systems. Wang et al. [90] used this strategy to introduce carboxyl groups, which synergized with the inherent amino groups of chitosan to further enhance the Zn^2+^ migration number and mechanical strength, while simultaneously lowering the desolvation energy barrier and demonstrating excellent interfacial stability.

**Figure 6:**
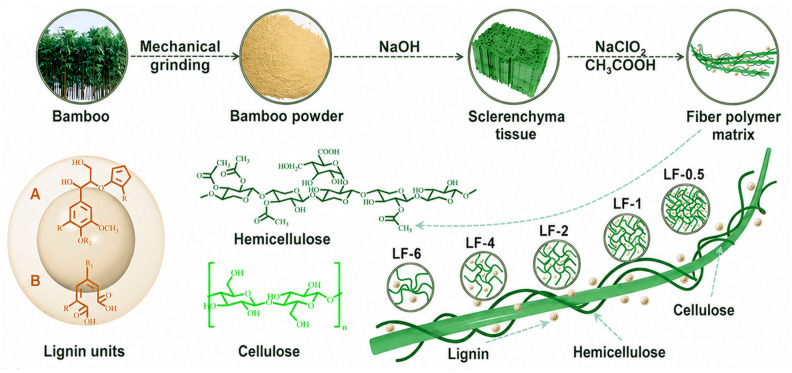
Schematic of bamboo processing and gradient lignin modification via controlled NaClO_2_ treatment, enabling simultaneous delignification, carboxylation, and mesoporous network formation. Reproduced from Ref. [91] with permission from Elsevier B.V., copyright 2026.

Leveraging the modifiable nature of phenolic and hydroxyl groups on its aromatic backbone, lignin is often intrinsically functionalized through chemical derivatization during the prepolymerization stage. While retaining its high thermal stability, this approach allows for the simultaneous optimization of surface polarity, pore structure, and interfacial compatibility via multiple pathways, including esterification, chlorination, and sulfonation. Modification via maleic anhydride esterification introduces unsaturated polar groups into the lignin molecular chain, enhancing surface polarity and affinity for electrolytes. For example, Yerkinbekova et al. reacted alkali-treated lignin with maleic anhydride to convert some hydroxyl groups into maleate ester groups; The modified lignin was blended with PAN and other materials, followed by in situ cross-linking via UV–electrospinning to prepare nanofiber membranes [41]. Chlorination and carboxylation can simultaneously regulate lignin composition and modify surface carboxyl groups to construct a mesoporous three-dimensional network. As illustrated in Figure 6, He et al. [91] treated bamboo nanofibers with sodium hypochlorite to achieve both partial selective removal of lignin and surface carboxylation. By leveraging the carboxylate coordination effect to promote uniform Zn^2+^ deposition, while relying on the aromatic backbone to confer excellent anti-swelling properties to the separator, this approach is well-suited for aqueous zinc-ion battery applications. Furthermore, sulfation modification can directly endow lignin with excellent intrinsic polarity; Jia et al. directly utilized sulfated lignin containing sulfonic acid groups to prepare ultrathin separators via a dry fiberization process [40]. The prepared ultrathin separator exhibits significantly improved interfacial affinity, capable of inducing the formation of a stable passivation layer at the electrode interface, thereby reducing interfacial impedance and enhancing the long-term cycling stability of lithium-ion batteries, while also offering potential for green production and large-scale application.

#### 4.1.2. Functional Coating Strategies

Functional coating strategies involve constructing ceramic, carbon-based, or polymer protective layers on the surface of biomass-based membranes. By reinforcing the mechanical strength, thermal dimensional stability, and electrolyte compatibility of the base membrane at the interfacial level, these strategies further optimize the ion transport environment and suppress dendrite growth. They serve as a crucial supplementary reinforcement method beyond surface chemical modification, with the focus of coating modifications for different biomass-based membranes varying according to their inherent defects.

Functional coatings for cellulose-based membranes primarily consist of ceramic composite coatings and polymer coatings, with the core objective of addressing their shortcomings in thermal stability and mechanical strength while simultaneously enhancing ion transport and dendrite suppression capabilities.

**Figure 7:**
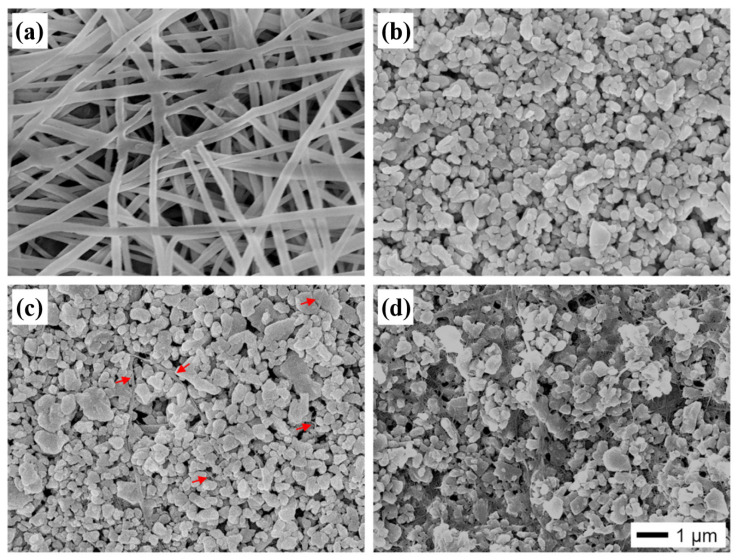
Top-view SEM images of Al_2_O_3_/nanocellulose (NC)-coated nonwoven separators with different NC contents. (**a**) Bare PVdF/PAN separator, (**b**) Al_2_O_3_/NC-0, (**c**) Al_2_O/NC-3, and (**d**) Al_2_O_3_/NC-7. Red arrows in (**c**) indicate nanocellulose fibers. All images share the same magnification. Reproduced from Ref. [92] according to the Creative Commons Attribution 4.0 International License.

Ceramic coatings, with their high thermal stability and mechanical strength, can effectively suppress high-temperature shrinkage of cellulose-based membranes and enhance interfacial stability. As illustrated in Figure 7, Shin et al. [92] employed a water-based doctor blade coating process to form hybrid metallogel coatings on the surface of cellulose nonwoven membranes by incorporating Al_2_O_3_ nanoparticles into the biomass gel network, significantly improving electrolyte adsorption capacity and ionic conductivity while effectively suppressing high-temperature thermal shrinkage and lithium dendrite growth.

Huang et al. [93] functionalized a bacterial cellulose separator with ZrO_2_ nanoparticles to construct metal-incorporated gel hybrid materials (metallogels), where metal oxides are incorporated into the biomass gel network. This approach effectively suppressed zinc dendrite growth, enabling the Zn||Zn symmetric cell to cycle stably for over 4500 h at 0.5 mA cm^−2^, and the full cell to maintain a capacity retention rate exceeding 92% after 1000 cycles at 5 A g^−1^.

**Figure 8:**
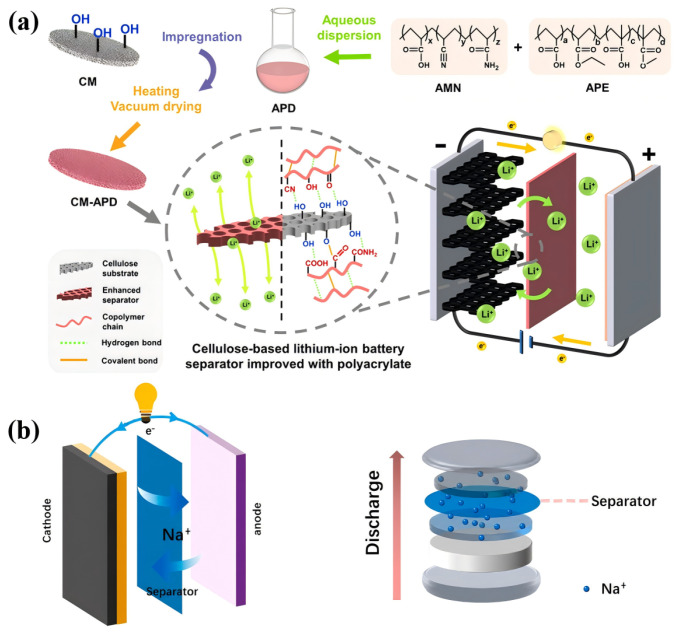
(**a**) Schematic illustration showing the preparation of a cellulose-based lithium-ion battery separator improved by aqueous polyacrylate dispersion (APD) via the impregnation method. The functional polyacrylate layer enhances the thermal stability, electrolyte wettability, and interfacial compatibility of the cellulose membrane. (**b**) The composite design combines the high porosity and electrolyte affinity of cellulose with the mechanical reinforcement and thermal stability of the PAN/Al_2_O_3_ nanofiber skins. Panel (**a**) is reproduced from Ref. [94] with permission from the American Chemical Society, copyright 2025. Panel (**b**) is reproduced from Ref. [96] with permission from Elsevier B.V., copyright 2026.

Polymer coatings, with their excellent flexibility and interfacial compatibility, form a uniform protective layer on the surface of cellulose-based membranes, improving electrolyte wetting and broadening the electrochemical stability window. As illustrated in Figure 8, Gong et al. [94] used a polyacrylate aqueous dispersion to construct a uniform protective layer on the surface of the cellulose membrane, broadening the electrochemical stability window to 5.29 V while significantly enhancing electrolyte adsorption capacity and ionic conductivity. Kim et al. [95] used a roll-to-roll gravure coating process to apply a PVDF polymer layer onto the surface of a cellulose-based separator, effectively enhancing the separator’s thermal stability while maintaining good pore structure and ionic transport properties.

Organic–inorganic metallogel hybrid coatings have emerged as one of the most promising research directions due to their combination of the thermal stability of ceramics and the flexibility of polymers. While pure ceramic coatings can improve thermal stability, they are highly brittle and exhibit poor interfacial compatibility, which can lead to coating delamination or increased interfacial resistance; conversely, pure polymer coatings improve flexibility but have limited thermal stability and insufficient control over dendrite growth. Organic–inorganic metallogel hybrid coatings effectively overcome these limitations by synergistically combining the advantages of both materials.

**Figure 9:**
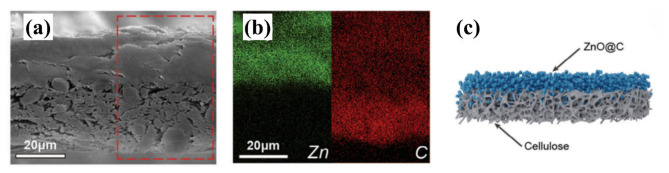
(**a**,**b**) Cross-sectional SEM images and (**c**) schematic model of the ZnO@C/cellulose separator. The red dashed box in panel (**a**) highlights the uniform ZnO@C coating layer on the cellulose substrate. The uniform ZnO@C coating layer (~7.9 µm) on the cellulose substrate enables in situ formation of a Li–Zn alloy layer during cycling, which effectively regulates lithium deposition and suppresses dendrite growth. All images share the same magnification. Reproduced from Ref. [97] according to the Creative Commons Attribution 4.0 International License.

Zhou et al. [96] designed a sandwich-structured coating with PAN/Al_2_O_3_ double-sided coating on cellulose, which significantly enhanced the mechanical strength and sodium-ion migration capacity of the separator, reduced interfacial resistance, and optimized high-rate performance. As illustrated in Figure 9, Chen et al. [97] in situ constructed a ZnO@C composite modified layer. By leveraging the lithium-affinity of ZnO to form a Li–Zn alloy layer and combining it with the conductive synergy of the carbon layer, they precisely controlled the direction of lithium deposition. This created a stable interfacial protective layer on the surface of the cellulose-based membrane, enabling the symmetric cell to achieve a cycle life exceeding 4500 h, demonstrating promising dendrite suppression and enhancing interfacial stability.

The core of coating modification for chitosan-based membranes lies in specifically addressing their inherent defects—such as moisture absorption and interfacial corrosion—and enhancing their cycling stability and environmental adaptability through interfacial regulation. While ceramic coatings alone can effectively block moisture penetration and improve thermal stability, they are inherently brittle and exhibit weak interfacial bonding with the chitosan matrix, making them prone to cracking and peeling under long-term cycling or mechanical stress [98]. Currently, polymer coating remains the mainstream strategy for modifying chitosan-based membranes. Polymer coatings exhibit good interfacial compatibility with chitosan, effectively suppressing substrate moisture absorption and reducing interfacial side reactions. For example, Hu et al. [99] used an impregnation method to construct a polydopamine (PDA) composite coating on the surface of a chitosan-based membrane. By leveraging strong hydrogen-bond networks formed between amino and catechol groups, they in situ regulated the interfacial microenvironment, effectively suppressing zinc corrosion and hydrogen evolution side reactions, and significantly improving the cycling life of symmetric cells and the capacity retention of full cells. Zhang et al. [100], on the other hand, coated the surface of chitin nanofiber membranes with a fluorinated polymer protective layer, effectively suppressing the moisture-absorption defects of the substrate and enhancing the compatibility at the electrode/separator interface, thereby demonstrating excellent cycling stability in lithium-ion battery systems. Although polymer components can provide flexibility and strong interfacial bonding, they have poor moisture resistance; ceramic components can provide excellent moisture-barrier properties. Forming an organic–inorganic composite coating can simultaneously address the two core issues of brittleness and insufficient moisture resistance. Although current research on chitosan-based membranes is still relatively limited, it shows great potential for achieving synergistic improvements in mechanical stability and ionic transport performance.

Coating modification of lignin-based substrates is still in its early stages of research, with the primary focus on using coatings to compensate for their inherent defects and expand their functional capabilities; there remains ample room for systematic exploration in this area. Compared to cellulose and chitosan, research on the coating modification of lignin-based membranes remains relatively limited. Systematic exploration of thermal barrier protection, interface modification, and multilayer composite structures through the construction of thin layers of ceramics or functional polymers is still in its infancy and can serve as an important research direction for the functional modification and structural design of biomass membranes in the future.

The design philosophy underlying functional coating strategies is to selectively address the primary defects of the biomass base membrane while preserving its intrinsic advantages (high porosity, hydrophilicity, and thermal stability). Ceramic coatings (e.g., Al_2_O_3_, ZrO_2_) primarily enhance thermal dimensional stability and mechanical puncture resistance, making them particularly effective for high-safety lithium-ion batteries under thermal runaway conditions. However, pure ceramic layers tend to be brittle, increase interfacial resistance, and may delaminate under long-term cycling or mechanical stress.

Polymer coatings (e.g., PVDF, polyacrylate, PDA, fluorinated polymers) improve flexibility, electrolyte wettability, and interfacial compatibility, and are especially useful for suppressing moisture absorption in chitosan-based membranes or enhancing high-voltage stability. Their main limitations are relatively lower thermal stability and weaker ability to suppress dendrite growth compared with ceramic coatings.

Organic–inorganic hybrid (metallogel) coatings combine the thermal robustness of ceramics with the flexibility and strong interfacial bonding of polymers, offering the best overall balance for most advanced applications (including lithium-metal, sodium-ion, and aqueous zinc-ion batteries). The trade-off is increased processing complexity and the need for careful optimization of the inorganic-to-organic ratio to avoid excessive resistance or reduced porosity.

In practice, the selection of coating strategy should be guided by the dominant failure mode of the target battery system. For high-safety lithium-ion batteries where thermal runaway is the primary concern, ceramic or hybrid coatings are generally preferred. For flexible devices or aqueous systems such as zinc-ion batteries that require strong interfacial regulation and moisture resistance, polymer or hybrid coatings are more suitable. When high-energy-density metal anodes demand simultaneous dendrite suppression and thermal stability, hybrid metallogel coatings. This principle-driven matching of coating chemistry to base-membrane defects and battery operating conditions provides a rational framework beyond mere case-by-case listing.

### 4.2. Multicomponent Composites and Hybridization

Building upon the effective optimization of membrane interfacial compatibility and electrolyte hydrophilicity through surface modification, multi-component composites and hybridization strategies can further achieve a significant leap in the overall performance of biomass-based membranes at the bulk structural level. This strategy employs methods such as blending, cross-linking, and inorganic hybridization/layer-by-layer self-assembly to deeply couple biomass fibers with polymeric materials and inorganic nano-functional particles at the molecular scale, thereby constructing a network of synergistic mechanisms. While preserving the matrix’s highly porous and interconnected structure, this approach simultaneously enhances the membrane’s mechanical toughness, thermomechanical stability, and ionic regulation capabilities, achieving synergistic improvements in mechanical, thermal, and electrochemical performance.

#### 4.2.1. Blending Strategy

Blending strategies involve methods such as solution blending and electrospinning to uniformly disperse biomass components and flexible polymers at the molecular scale. This effectively addresses the shortcomings of single-biomass materials—such as high brittleness and insufficient mechanical properties—while maximizing the retention of their natural hydrophilicity and porous structure. Based on the types of blending components and functional objectives, these modification approaches can be categorized into two types: biomass–biomass blending and biomass–polymer blending.

Biomass–biomass blending relies on non-covalent interactions between natural components to synergistically optimize the pore structure of the membrane and the wetting properties of the electrolyte without the need for additional additives. For example, Serra et al. [101] used a solution blending method to composite algal cellulose with soybean isolate protein, forming a uniform interpenetrating network via intermolecular hydrogen bonds. Without the need for additional cross-linking agents, they simultaneously enhanced ionic conductivity and thermomechanical stability solely through the polar interactions of natural components. However, this strategy relies on weak non-covalent interactions, resulting in limited interfacial bonding strength; phase separation is prone to occur during long-term electrolyte immersion or cycling, and its long-term stability requires further validation.

**Figure 10:**
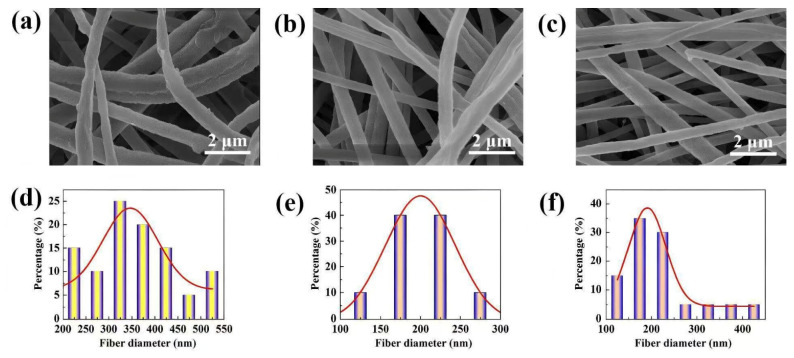
SEM images and fiber diameter distributions of electrospun PI/lignin nanofiber separators with different PI/L blending ratios. (**a**–**c**) SEM images of PI/L nanofiber membranes with blending ratios of 10/1, 10/2, and 10/3, respectively; (**d**–**f**) corresponding fiber diameter distributions of the membranes shown in panels (**a**–**c**), respectively. Reproduced from Ref. [86] with permission from Elsevier B.V., copyright 2023.

Biomass–polymer blending strategies can, to some extent, reinforce the mechanical properties of biomass-based separators and broaden the electrochemical window by introducing flexible or rigid polymer segments. However, the widespread thermodynamic incompatibility between biomass and synthetic polymers often leads to significant phase separation. While this creates free volume for ion transport, it also poses challenges such as weakened interfacial bonding, reduced mechanical strength, and insufficient long-term stability. For example, in cellulose–polymer blends, Mohamadzade et al. [102] blended cellulose with PVDF to construct a regular pore structure via microphase separation, achieving extremely high lithium-ion transference numbers and a wide electrochemical stability window, thereby significantly enhancing the battery’s high-voltage compatibility and rate performance. However, due to the high Flory–Huggins interaction parameters between cellulose and PVDF, their thermodynamic incompatibility leads to significant phase separation. While this facilitates the formation of free volume for ion transport, it also causes the mechanical strength of the separator to decrease significantly with increasing cellulose content (from 9.5 MPa to 2.1 MPa). In lignin–polymer blends, as illustrated in Figure 10, Song et al. [86] blended sulfonated lignin with polyimide via electrospinning, utilizing π–π stacking and hydrogen bonding to construct an interconnected three-dimensional nanonetwork. This approach combines excellent thermal and dimensional stability with long-cycle capacity retention, resulting in overall performance significantly superior to that of commercial polyolefin separators. However, pure polyimide electrospun membranes inherently exhibit low mechanical strength (approximately 5 MPa). Although the addition of lignin provides some improvement, interfacial compatibility still requires further optimization, and high lignin content may lead to uneven fiber diameters and a non-uniform pore structure.

#### 4.2.2. Crosslinking Strategies

Crosslinking strategies refer to the construction of a robust three-dimensional crosslinked network between biomass and polymer components through physical, chemical, UV light, or hot-press crosslinking methods, thereby achieving bulk stiffness reinforcement and simultaneously enhancing the membrane’s mechanical strength, thermal dimensional stability, and resistance to electrolyte swelling. Based on differences in bonding mechanisms, these strategies can be classified into two categories: physical crosslinking and chemical crosslinking.

Physical crosslinking primarily relies on non-covalent interactions, such as hydrogen bonding and electrostatic ionic complexation, to form a network structure. This process is mild and can balance pore characteristics with structural stability. Deng et al. [103] combined electrospinning with thermal imidization to prepare a cellulose/carboxylated polyimide composite separator. By utilizing intermolecular hydrogen bonds to form a three-dimensional interpenetrating network, they significantly improved tensile strength without the addition of crosslinking agents, while achieving near-zero thermal shrinkage at 150 °C, balancing electrolyte wettability and thermal safety performance. Arif et al. [104] achieved ionic cross-linking through polyelectrolyte complexation, forming a dense network based on electrostatic interactions between positive and negative charges on chitosan and carboxymethyl chitosan. The abundant surface active sites effectively enhanced the selective transport of zinc ions and suppressed dendrite growth and evolution.

Chemical cross-linking, based on covalent bonds, can construct high-strength, highly stable cross-linked networks, fundamentally enhancing the structural integrity and electrochemical stability of the separator. Trano et al. [105] employed epoxy-based covalent crosslinking to bond pre-oxidized lignin with polyethylene glycol diglycidyl ether, forming a dense three-dimensional network. This significantly improved the mechanical strength and ionic conductivity of the separator, with cycling performance superior to that of commercial separators, providing a viable solution for biomass-based electrolyte separators in potassium-ion batteries.

#### 4.2.3. Inorganic Hybridization/Layered Self-Assembly

Inorganic hybridization and layer-by-layer self-assembly serve as core strategies for breaking through the performance limitations of purely organic biomass-based separators, effectively addressing their inherent bottlenecks of low ionic transport efficiency and insufficient interfacial control. This strategy involves introducing inorganic nano-functional components such as SiO_2_, Al_2_O_3_, MOFs, and nanotubular fillers to construct a well-organized organic-inorganic composite network. This approach simultaneously enhances ion selectivity, salt dissociation efficiency, and dendrite suppression capabilities, ultimately achieving multidimensional synergistic optimization of the separator’s mechanical, thermal, and electrochemical properties.

Based on their modification mechanisms and structural regulation characteristics, inorganic hybridization and layer-by-layer self-assembly can be categorized into two major types: inorganic nanoparticle hybridization and layer-by-layer self-assembly interface modification. These approaches enhance performance at the bulk structural and surface interface levels, respectively, to meet diverse application requirements.

**Figure 11:**
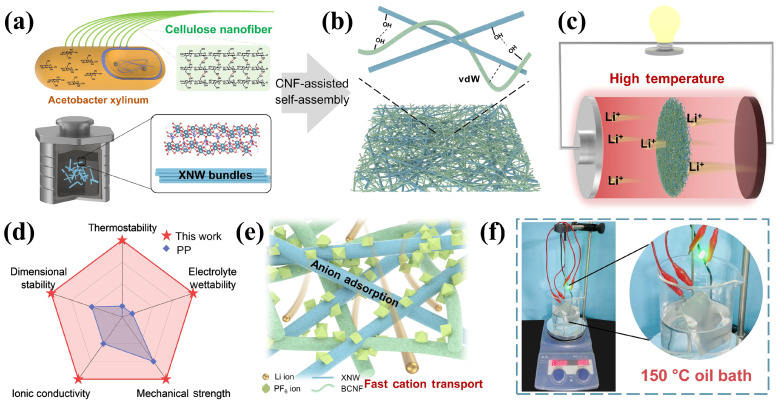
Schematic illustrations of the cellulose nanofiber (CNF)-assisted self-assembly of Xonotlite nanowires (XNW) into a porous double-network CNF-XNW separator (**a**,**b**) fabrication process; (**c**–**f**) Regulated Li^+^ transport behavior, high-temperature operation, and thermal stability of the separator. Reproduced from Ref. [106] according to the Creative Commons Attribution 4.0 International License, copyright 2025, published by Tsinghua University Press.

Inorganic nanoparticle hybridization focuses on optimizing bulk structure. By leveraging the synergistic interaction at the interface between the biomass matrix and inorganic particles, it precisely controls the gel network topology and surface physicochemical properties, balancing thermal stability with ionic transport efficiency. As illustrated in Figure 11, Yang et al. [106] utilized hydrogen bonds and van der Waals forces to construct a highly connected three-dimensional pore network, achieving inorganic hybridization between cellulose nanofibers and Xonotlite nanowires (XNWs). By leveraging the synergistic effects at the organic-inorganic interface, they simultaneously optimized ionic conductivity and high-temperature thermal stability, significantly enhancing the battery’s rate capability and cycling performance, thereby providing a green separator solution for high-safety lithium-ion batteries under extreme conditions.

Layer-by-layer self-assembly focuses on precise interfacial control. By constructing ordered multilayer structures through alternating electrostatic deposition, it enables fine-tuning of surface charge distribution and pore characteristics, thereby enhancing interfacial stability and ion sieving capacity. Yang et al. [107] utilized layer-by-layer self-assembly technology to electrostatically deposit chitosan and polystyrene sulfonate onto a substrate surface. By forming ordered multilayer structures through the complexation of positive and negative charges, they precisely controlled pore structure and surface charge, significantly optimizing ionic conductivity, thermal stability, and cycling performance under electrolyte depletion conditions, thereby providing a thin, green, and functional separator solution for high-safety, long-life lithium-metal batteries.

Notably, the introduction of nanotubular inorganic fillers can further enhance the functionality of the hybrid system. By synergistically regulating functional group interactions and pore structures, both ionic transport and thermal safety performance are simultaneously improved. Song et al. [108] introduced halloysite nanotubes into a polyimide/lignin oxide-based membrane for inorganic hybridization. By leveraging hydrogen bonds and the tubular structure to construct a uniform three-dimensional network, they effectively improved the ionic conductivity and thermal dimensional stability, achieving superior long-cycle performance compared to commercial PP separators and pure PI@OL separators, thereby providing a biomass-based inorganic hybrid separator solution for high-safety, long-life lithium metal batteries.

The underlying design principle of multicomponent strategies is to achieve synergistic enhancement of mechanical toughness, thermal stability, and ion-transport efficiency while minimizing phase separation and loss of porosity. Blending improves processability and mechanics but risks long-term interfacial instability; chemical crosslinking provides permanent network strength at the cost of reduced swelling and ion mobility if overdone; inorganic hybridization offers the highest multifunctional gains but requires careful dispersion control.

### 4.3. Fine-Tuning of Structural Design

Refined structural design represents an advanced strategy for enhancing the performance of biomass-based separators, following surface modification and multi-component composites. It can overcome the inherent limitations of traditional disordered-pore separators regarding ion transport and dendrite suppression. By constructing gradient pores, vertically ordered channels, and biomimetic multi-level topological structures, it is possible to optimize ion transport behavior while maintaining high porosity and structural integrity, thereby achieving synergistic improvements in mass transfer, dendrite suppression, and thermal stability.

#### 4.3.1. Gradient Pores and Multidimensional Gradient Structure Design

Gradient pore design is a key approach for fine-tuning the structure of cellulose-based separators. By leveraging techniques such as non-solvent-induced phase separation, concentration gradient control, and template-assisted self-assembly, it is possible to create a non-symmetrical pore size distribution characterized by a dense surface layer and a porous interior. This configuration optimizes ion transport pathways, enhances flux uniformity and ion selectivity, effectively addresses the shortcomings of multi-component composites in the ordered regulation of macroscopic pore channels, and achieves synergistic benefits in directed ion transport and long-term dendrite suppression.

**Figure 12:**
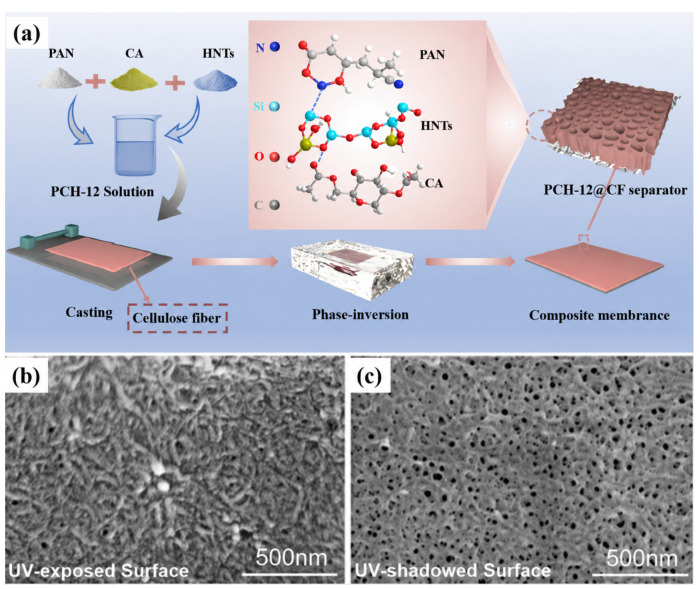
(**a**) Fabrication and gradient pore structure of a cellulose-based separator via NIPS. The PCH-12@CF separator exhibits a dense surface layer and a loose interior with HNTs for uniform Zn^2+^ transport and dendrite suppression. (**b**,**c**) SEM images of the gradient pore structure in the PCGU aerogel separator. (**b**) UV-exposed side (2–6 nm nanopores); (**c**) Non-exposed side (15–40 nm mesopores). The asymmetric dual-network enables selective ion sieving. Panel (**a**) is adapted from Ref. [109] with permission from Elsevier B.V., copyright 2025. Panels (**b**,**c**) are adapted from Ref. [110] with permission from Elsevier B.V., copyright 2026.

Phase separation coupled with inorganic hybridization is the mainstream approach for constructing continuous gradient pore channels. As illustrated in Figure 12, Jiao et al. [109] used cellulose as a substrate and combined phase separation with HNT nanotube modification to construct a gradient pore network with a dense surface and a porous interior. The gradient structure enables the directed regulation of ion flux. Coupled with the synergistic action of Lewis acid sites on the inorganic fillers, this approach simultaneously enhances mechanical and ionic conductivity, achieving long-term suppression of zinc dendrites. This provides a structurally refined, green solution for high-safety, long-life aqueous zinc-ion batteries.

Physical assembly strategies can easily achieve the controlled construction of cross-scale gradient pore morphologies. Huang et al. [110] prepared an asymmetric gradient-pore aerogel separator (PCGU) based on regenerated cellulose through the dissolution-regeneration coupled with UV-induced PNIPAM polymerization. The precisely controlled gradient pore structure regulates ion transport behavior, while the dual-network architecture enhances mechanical stability. This approach demonstrates excellent long-cycle performance and capacity retention in zinc-based energy storage systems, providing a structurally refined, green separator solution for high-safety, long-life zinc-ion hybrid supercapacitors.

**Figure 13:**
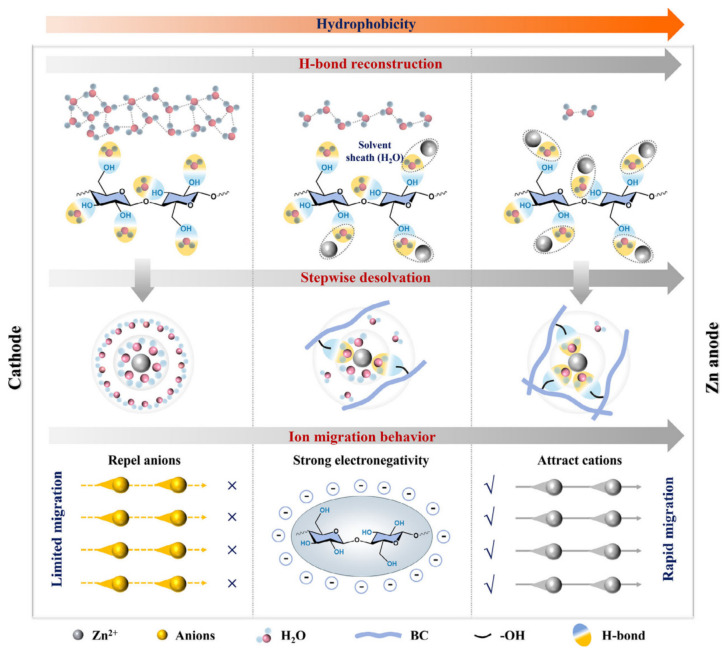
Hydrophobicity gradient mechanism in an ultrathin cellulose separator. The SiO_2_-induced gradient enables stepwise Zn^2+^ desolvation, H-bond reconstruction, and Cl^−^ shielding for stable seawater-based batteries. Reproduced from Ref. [111] with permission from Wiley-VCH GmbH, copyright 2025.

Gradient regulation can also be extended from pore morphology to multidimensional synergistic design of surface wettability. As illustrated in Figure 13, Dong et al. [111] employed hydrophobic nanoparticle impregnation to construct a hydrophilic–hydrophobic gradient structure on a cellulose-based membrane. The gradient interface establishes a stepwise desolvation pathway for Zn^2+^, and synergizes with electrostatic shielding effects to suppress corrosion and hydrogen evolution reactions. More importantly, the high liquid uptake enables the formation of stable ionic gel and hydrogel electrolyte interfaces that suppress dendrite growth within the gel network, making it suitable for the harsh operating environment of seawater electrolytes.

#### 4.3.2. Construction of Vertically Ordered Ion Channels

While gradient pore structures focus on passively guiding ion migration, the design of vertically ordered channels centers on reducing pore tortuosity to construct nearly straight ion transport pathways, effectively mitigating concentration polarization issues under high current densities. The natural anisotropic growth characteristics of biomass materials confer unique structural advantages for constructing vertical channel separators with low tortuosity.

Directional freezing technology can be used to biomimeticly fabricate vertically aligned, ordered ion channels. Ma et al. [112] drew inspiration from plant vascular structures, using nanocellulose as a matrix to construct axially vertical ion channels via oriented freezing. This low-bend-radius architecture combines high modulus with ionic conductivity, homogenizes the interfacial electric field, and fundamentally suppresses the nucleation and growth of zinc dendrites, providing a structurally refined, green solution for high-safety, long-life aqueous zinc-ion batteries.

Chemically modified doping, on the other hand, induces the spontaneous formation of vertical penetrating channels within the polymer matrix. Tian et al. [113] subjected bacterial cellulose to cyanoethylation modification; following trace doping, this induced the formation of vertical channels within a polyethylene oxide (PEO) matrix. This configuration suppresses polymer crystallization, optimizes ion migration kinetics, and effectively enhances electrolyte ionic conductivity and cycling stability, providing a sustainable modification pathway for biomass-based solid electrolytes.

Overall, the core design principles of gradient asymmetric pores and vertically ordered ion channels both lie in reconstructing the disordered and random gel network topology of traditional biomass-based separators. Such refined designs integrate biomimetic concepts such as plant vascular transport and interfacial gradient screening, upgrading separator modification from two-dimensional interfacial modification to precise three-dimensional bulk control, thereby achieving a technological leap from passive physical barrier to active induction of ion behavior. When synergistically coupled with the surface modification and multi-component composite modification strategies discussed earlier, an integrated enhancement system combining chemical modification, component hybridization, and structural fine-tuning can be established. This provides a solid theoretical and technological foundation for the large-scale application of high-energy-density, safe, and stable aqueous and all-solid-state energy storage batteries.

Refined structural design (gradient pores, vertical channels) follows the principle of actively directing ion flux rather than passively relying on random porosity. The trade-off is increased fabrication complexity versus superior rate performance and dendrite suppression under high current densities.

## 5. Research on Performance Adaptability for Different Battery Systems

The preceding sections systematically discussed the modification evolution of biomass-based separators, ranging from surface modification and multi-component composites to fine-tuned structural design, providing diverse technical pathways for constructing high-performance, sustainable key battery components. However, the practical application value of separators ultimately depends on their performance adaptability and system-level long-term stability under complex electrochemical environments. Since different battery systems (such as high-voltage lithium-ion batteries, highly reactive aqueous zinc batteries, or lithium–sulfur batteries with polysulfide shuttling) have distinct requirements regarding ion transport kinetics, interfacial compatibility, and chemical stability, establishing a deep structure-property relationship between “modification strategies–application scenarios–performance” is particularly important. This chapter aims to expand the research scope from the “intrinsic modification” of materials to “system-level validation”. It focuses on reviewing the performance of functionalized biomass-based separators across multiple scenarios (high safety, high rate capability, and long cycle life) in conventional lithium-ion batteries (LIBs), and further explores their adaptation mechanisms and unique advantages in emerging energy storage systems such as sodium/potassium-ion batteries, lithium–sulfur batteries, and solid-state batteries. Through a comparative analysis and tabular summary of key electrochemical metrics, this chapter will comprehensively evaluate the core competitiveness and challenges faced by biomass-based separators as they progress toward practical industrial applications.

### 5.1. Performance in Lithium-Ion Batteries

As the most mature and mainstream energy storage system currently available, the performance of lithium-ion batteries relies heavily on the comprehensive performance of separators in terms of thermal safety, rate capability, and cycle life. Through surface modification, multi-component composites, and refined structural design, the mechanical toughness, thermal and dimensional stability, and ionic conductivity of biomass-based separators have been significantly improved, providing a solid foundation for practical battery applications. However, different application scenarios impose distinct requirements on separators: high-safety batteries prioritize the suppression of thermal shrinkage and dendrite formation; high-power batteries seek high ionic conductivity and excellent rate capability; and long-life batteries require long-term cycling stability. This section will systematically review the adaptation mechanisms and actual performance of specific modification strategies across three scenarios—high-safety, high-power, and long-life—using representative case studies.

#### 5.1.1. High-Safety Type

The core requirements for high-safety lithium-ion batteries are that the separator must possess extremely low thermal shrinkage, excellent flame-retardant properties, and strong dendrite suppression capabilities, thereby reducing the risks of thermal runaway and short circuits at the source. Due to their abundant polar functional groups and high thermal stability (thermal decomposition temperatures typically exceeding 300 °C), biomass fibers can significantly enhance battery safety under extreme operating conditions through cross-linking, inorganic hybridization, and structural refinement strategies.

**Figure 14:**
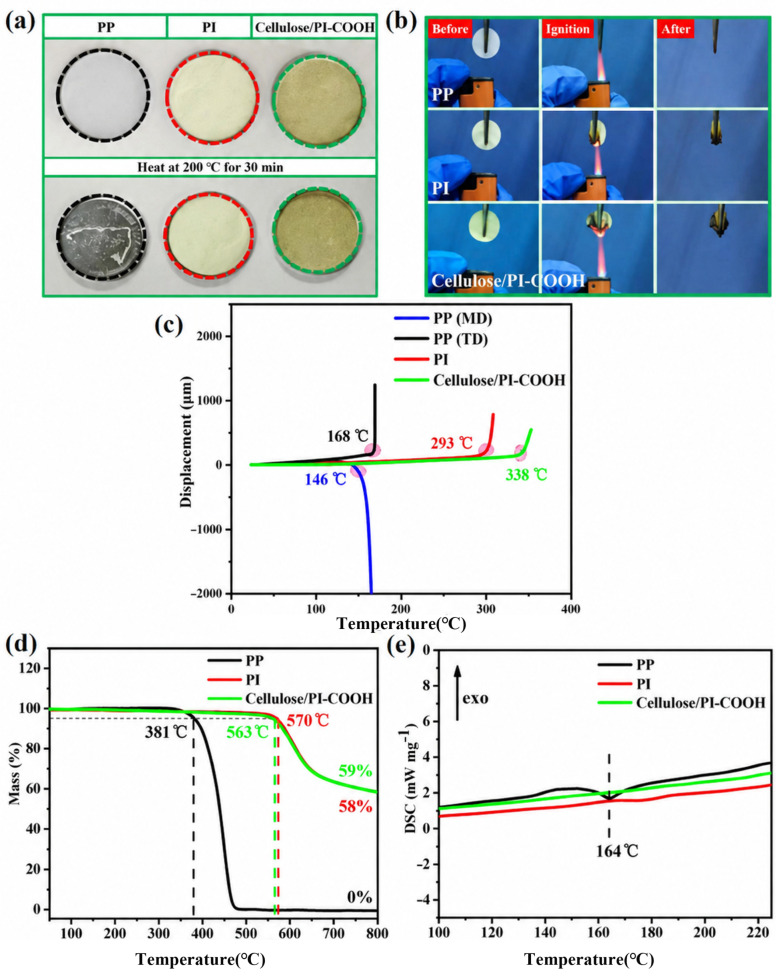
(**a**) Digital photographs of PP, PI, and Cellulose/PI-COOH separators before and after being heated at 200 °C for 30 min; (**b**) flame retardant performance tests; (**c**) TMA curves of the separators; (**d**) TG curves and (**e**) DSC curves of PP, PI, and Cellulose/PI-COOH separators. Reproduced from Ref. [103] with permission from Elsevier B.V., copyright 2021.

Regarding thermal dimensional stability and mechanical reinforcement, molecular-level crosslinking and high-performance polymer composites are the primary strategies. As illustrated in Figure 14, Deng et al. [103] utilized dense hydrogen bonds between cellulose and carboxylated polyimide (PI–COOH) to construct a three-dimensional interpenetrating network. The tensile strength of this composite separator reached five times that of a pure PI membrane, and it maintained a thermal shrinkage rate of nearly 0% at 150 °C, effectively preventing anode-cathode contact at high temperatures and ensuring the long-term cycling stability of LiFePO_4_/Li batteries. To further push the limits of flame retardancy under extreme high temperatures, Ren et al. [114] uniformly anchored flame-retardant groups within the cellulose network via a cross-linking reaction. This separator not only exhibits excellent self-extinguishing properties but also achieves zero thermal shrinkage under extreme baking conditions as high as 240 °C, cutting off the propagation pathways of thermal runaway from both physical and chemical dimensions.

In terms of suppressing metal dendrite penetration and optimizing interfacial mass transfer, the synergistic effect of structural refinement and active fillers demonstrates unique advantages. To address the micro-short-circuiting issues caused by non-uniform deposition of metal anodes, Jiao et al. [109] combined halloysite nanotubes (HNTs) to construct a gradient-porous separator with a loose interior and a dense surface. While maintaining zero shrinkage at 150 °C, this design not only significantly improved ionic conductivity but also achieved excellent dendrite suppression under high current (10 mA cm^−2^). Furthermore, Fu et al. [115] innovatively introduced a micro-coiled structure layer encapsulating the flame-retardant DBDPE onto the surface of a cellulose-based membrane. This unique nanosheet physical barrier not only homogenizes ion flux to suppress lithium dendrite tip growth but also responsively releases the flame retardant during abnormal temperature rises, significantly delaying the onset of thermal runaway in Li||Li symmetric cells.

Despite these advances in thermal dimensional stability and flame retardancy, several challenges remain for biomass-based separators in high-safety lithium-ion batteries. Long-term performance under realistic abuse conditions (such as nail penetration, overcharge, and thermal runaway propagation) is still insufficiently validated. In addition, many crosslinking or hybrid strategies that achieve zero thermal shrinkage may compromise ionic conductivity or increase interfacial resistance at high rates. Scalable, low-cost manufacturing of flame-retardant biomass separators that maintain both high mechanical strength and excellent ion transport under extreme temperatures continues to be a critical barrier to commercialization.

#### 5.1.2. High-Power Type

Unlike the high-safety type, which focuses on physical barriers under extreme conditions, the core challenge of high-power lithium-ion batteries lies in the fact that, during high-rate charging and discharging, the liquid-phase mass transfer rate of ions often lags behind solid-phase electron transfer, thereby triggering severe concentration polarization and interfacial lithium plating. Therefore, the separator must possess extremely low mass transfer resistance (low tortuosity) and excellent electrolyte affinity. Biomass-based materials, with their naturally abundant polar functional groups and highly tunable macroscopic topological structures, demonstrate irreplaceable kinetic advantages in overcoming the mass transfer bottlenecks of traditional porous separators.

**Figure 15:**
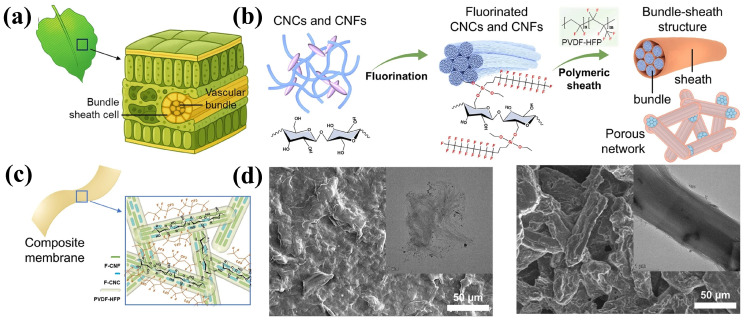
(**a**) Schematic of a natural plant vascular bundle (source of inspiration); (**b**,**c**) formation mechanism and biomimetic “bundle-sheath” architecture of the fluorinated nanocellulose/PVDF-HFP (FFP) composite membrane; (**d**) SEM and TEM images showing the morphology of the disordered control membrane and the highly ordered FFP membrane with low-tortuosity ion transport channels. Reproduced from Ref. [118] according to the Creative Commons Attribution 4.0 International License.

The highly polar network of biomass macromolecules plays a decisive role in reducing interfacial impedance and accelerating ion desolvation. Compared to traditional polyolefin separators, which are inherently hydrophobic and poorly wetted, the nanoporous regenerated cellulose separator prepared by Wang et al. [76] achieved extremely high electrolyte absorption due to the dense distribution of polar hydroxyl groups (–OH) on the cellulose framework, effectively accelerating the dissociation of the Li^+^ solvation shell, enabling the battery to maintain excellent capacity retention even at a high rate of 5 C. To further enhance interfacial kinetics, as illustrated in Figure 15, Zheng et al. [116] cross-linked cellulose nanofibers (CNFs) with carboxymethyl cellulose (mCMC) to construct an all-cellulose composite separator with an asymmetric Janus pore structure. This not only effectively suppressed the disordered migration of anions but also further accelerated the desolvation process of Li^+^. The full cell equipped with this separator still demonstrated excellent capacity utilization and cycling retention at an extremely high rate of 7 C, proving the immense potential of polar synergistic networks in overcoming the interfacial energy barrier for fast charging.

In terms of reducing the geometric tortuosity of macroscopic ion transport pathways, the refined design of vertical or oriented channels is a key strategy for achieving ultrafast mass transfer. Traditional disordered fiber networks are highly prone to causing detours and blockages in ion transport pathways, whereas the vertically aligned ion-channel cellulose composite separator customized by Liu et al. [117] significantly shortened the macroscopic migration distance of Li^+^ and homogenized the ionic flow through spatial confinement effects. Furthermore, inspired by plant vascular bundles, Gao et al. [118] developed a biomimetic vertical channel composite membrane (FFP) with a “bundle-sheath” structure. This refined design not only minimizes tortuosity through vertical channels but also promotes lithium salt dissociation via a PVDF-HFP sheath layer.

Although biomass-based separators with polar networks and low-tortuosity channels have demonstrated excellent rate capability, challenges persist in high-power applications. Maintaining uniform ion flux and low polarization under ultra-high current densities over thousands of cycles remains difficult. The trade-off between high porosity (necessary for fast ion transport) and mechanical robustness under electrode volume changes is not yet fully resolved. Furthermore, most reported high-rate performances are obtained in laboratory coin cells; validation in large-format pouch or cylindrical cells with lean electrolytes is still limited.

#### 5.1.3. Long-Cycle-Life Type

Capacity decay in high-energy-density lithium-ion batteries during long-cycle operation primarily stems from continuous side reactions at the electrode/electrolyte interface, repeated rupture and thickening of the solid-electrolyte interphase (SEI), and the leaching and shuttling of cathode transition metal ions. To achieve long-life energy storage, the separator must transform from a passive “physical barrier” into an active “interface guardian.” As discussed in Chapter 4, biomass-based separators, through surface functional group modification and a robust, porous network design, demonstrate inherent advantages in forming a robust SEI film and eliminating harmful byproducts within the battery.

In stabilizing interfacial kinetics and geometric pore channels, highly polar networks and high-modulus frameworks play a synergistic supporting role. The abundant surface oxygen-containing functional groups in biomass materials are deeply involved in reshaping the solvation structure of the electrolyte, not only homogenizing the interfacial flux of cations but also inducing the formation of a dense, low-impedance, stable SEI/CEI film through specific adsorption. For example, Jia et al. [40] successfully induced the formation of a sulfur-rich CEI/SEI protective layer in a full cell by utilizing the abundant sulfonic acid groups in a lignin-based separator. The bacterial cellulose gradient porous separator developed by Liu et al. [117] homogenized ion transport through the synergistic effects of nanoconfinement and an ordered hydrogen-bond network. This refined structure is suggested to help maintain a more constant ionic flux during long-term cycling, thereby contributing to the suppression of interfacial side reactions, enabling the full cell equipped with this separator to maintain excellent long-term capacity performance even under the harsh conditions of a low N/P ratio.

**Figure 16:**
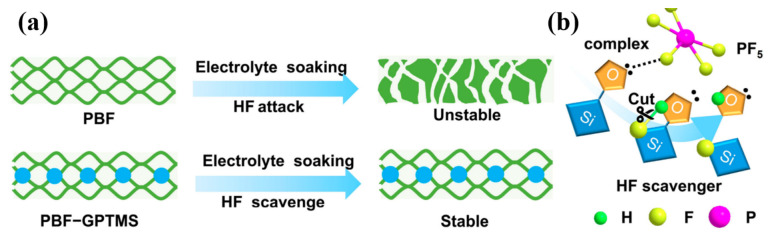
Schematic illustration of the HF scavenging mechanism by the silane-cross-linked cellulose separator. (**a**) Working conditions of PBF and PBF-GPTMS separators after electrolyte soaking, showing HF attack-induced instability in PBF and HF-scavenging-enabled stability in PBF-GPTMS. (**b**) HF-scavenging mechanism of GPTMS, where oxygen lone pairs in the Si–O–C structure complex with PF_5_ to suppress HF formation. Reproduced from Ref. [88] according to the Creative Commons Attribution 4.0 International License.

In addition to positive regulation at the interface, the biomass separator also plays a key role as a “chemical filter” during long-term cycling. When paired with high-voltage cathodes, the hydrolysis of lithium hexafluorophosphate (LiPF6) triggered by trace moisture in the electrolyte produces highly toxic and corrosive hydrofluoric acid (HF), which in turn causes transition metal ions (such as Mn^2+^ and Co^2+^) to leach out and migrate to the anode, poisoning the SEI film. Functionalized biomass-based separators, leveraging their high-density nucleophilic sites, can efficiently capture these harmful byproducts. Pereira et al. [119] utilized the dense hydroxyl network of nonwoven cellulose separators as an “ion sponge” to efficiently remove residual H_2_O and HF, resulting in a significant extension of the cycle life of commercial cylindrical and pouch batteries. To further enhance capture efficiency, As illustrated in Figure 16, Cui et al. [88] designed a silane-crosslinked cellulose separator that ingeniously leverages the lone pair of electrons on oxygen atoms within the Si–O–C structure to form strong complexes directly with PF5. This design cuts off the HF generation pathway at its source, significantly reducing the extent of corrosion at the anode interface. This active “chemical filtration” mechanism completely blocks the dual corrosion of both the anode and cathode by harmful substances, resulting in a dramatic improvement in the lifespan of the entire battery during long-term operation.

While functionalized biomass separators have shown strong ability to stabilize SEI/CEI layers and scavenge harmful species such as HF, long-term interfacial instability continues to limit cycle life in high-energy-density lithium-metal and high-voltage lithium-ion batteries. The progressive degradation of the biomass network itself under repeated charge–discharge, especially in the presence of transition-metal ions or highly reactive intermediates, requires further investigation. In addition, the long-term chemical stability of surface functional groups and the consistency of scavenging performance across different electrolyte formulations remain important open issues.

### 5.2. Exploration in Emerging Battery Systems

Although functionalized biomass-based separators have demonstrated outstanding overall performance in conventional lithium-ion batteries (LIBs), the pursuit of ultra-low costs for future grid-scale energy storage, as well as the demand for ultra-high energy density and intrinsic safety in aerospace and long-endurance power batteries, necessitates the development of emerging energy storage systems such as sodium/potassium-ion batteries (SIBs/KIBs), high-energy-density lithium–sulfur (Li-S) batteries, and all-solid-state batteries (SSBs) has become an inevitable trend. However, compared to the highly mature lithium-ion battery technology, the internal electrochemical environments of these next-generation battery systems are more complex and extreme. For example, the sluggish kinetics caused by large Na^+^/K^+^ ions, the severe polysulfide shuttling effect in Li-S batteries, and the extremely high solid–solid interfacial impedance and mechanical puncture risks in solid-state batteries all impose stringent demands on the gel network topology, chemical affinity, and mechanical stability of separators—demands that exceed the limits of traditional polyolefins. This section will systematically review and discuss the cutting-edge applications of biomass-based separators in the aforementioned emerging battery systems, focusing on how they leverage their unique cross-scale macroporous networks, high-density targeted anchoring sites, and rigid–flexible skeletal features to overcome mechanistic bottlenecks in these novel systems, while objectively assessing the new challenges they face on the path to practical application.

#### 5.2.1. Sodium/Potassium-Ion Batteries

Sodium-ion batteries (SIBs) and potassium-ion batteries (KIBs) are considered ideal candidates for large-scale grid energy storage due to their extremely low resource costs. However, the ionic radii of Na^+^ (1.02 Å) and K^+^ (1.38 Å) are significantly larger than that of Li^+^ (0.76 Å). This size effect results in substantial steric hindrance and extremely high desolvation energy barriers when these ions migrate through the narrow channels of conventional commercial polyolefin separators. This not only causes severe concentration polarization and sluggish reaction kinetics but also readily induces the growth of coarse, loose sodium/potassium metal dendrites, posing serious safety hazards. Faced with these inherent challenges posed by physical size, biomass-based separators demonstrate unparalleled adaptability due to their unique cross-scale porous topological networks and highly polar surfaces.

In overcoming the mass transfer barrier for large-sized Na^+^ and optimizing the electrolyte environment, the multi-scale pore channels and composite interfaces of biomass play a decisive role. Compared to hydrophobic polyolefin separators, which are prone to pore blockage, the matrix formed by interwoven biomass fibers naturally possesses more spacious ion transport channels. For example, Jo et al. [120] developed a cellulose–polyacrylonitrile (PAN)/alumina (Al_2_O_3_) composite separator. This design not only utilizes cellulose’s intrinsic three-dimensional, multi-scale pore structure and polar hydroxyl (–OH) network to eliminate steric hindrance for large Na^+^ ions but also ingeniously employs an Al_2_O_3_ coating to efficiently capture trace amounts of HF in the electrolyte (which is converted in situ to AlF_3_), thereby further purifying the ion transport environment. This synergistic effect of “physical topology + chemical scavenging” significantly increases the sodium ion mobility to 0.78, effectively homogenizing the Na^+^ flux and suppressing dendrite growth. The full cell equipped with this separator still exhibits excellent long-term cycling stability at a high rate of 10 C, completely reversing the disadvantage of poor rate performance in sodium batteries.

Compared to Na^+^, K^+^, with its larger radius, is more prone to inducing severe interfacial side reactions and coarse metal dendrites during deposition. To address this critical challenge in potassium batteries, Jia et al. [40] utilized a low-cost dry process to prepare a lignin-based ultrathin separator rich in sulfonic acid groups. This separator fully leverages the high modulus of the lignin aromatic backbone to construct a robust physical barrier that prevents large-sized potassium dendrites from penetrating the interface; more critically, the densely distributed polar sulfonic acid groups on its surface actively participate in regulating interfacial solvation, inducing the formation of a robust, sulfur-rich CEI/SEI layer. This high-quality interfacial passivation layer significantly reduces the desolvation energy barrier and resistance for large K^+^ ions crossing the interface, ensuring uniform potassium ion deposition under high current conditions. Thanks to this dual structural and chemical defense mechanism that combines rigidity and flexibility, the assembled potassium battery demonstrates excellent capacity utilization and long cycle life.

For sodium- and potassium-ion batteries, biomass-based separators have effectively addressed the steric hindrance of large Na^+^/K^+^ ions through multi-scale pore networks and polar surfaces. However, remaining challenges include insufficient compatibility with conventional carbonate-based electrolytes, which often leads to higher interfacial resistance and poorer long-term stability compared with ether-based systems. Dendrite growth of sodium and potassium metal under high areal capacity is still not completely suppressed. Scalable production of ultrathin, high-strength biomass separators specifically tailored for the larger ionic radii and different solvation structures of Na^+^ and K^+^ also requires further development.

#### 5.2.2. Lithium–Sulfur Batteries

Due to their extremely high theoretical specific energy (2600 Wh kg^−1^), lithium–sulfur (Li-S) batteries are considered an ideal choice for next-generation high-energy-density energy storage. However, their commercialization is severely hampered by the “shuttle effect” of the intermediate product, lithium polysulfides (LiPSs). Conventional commercial polyolefin separators, due to their inherent non-polarity, can only provide physical isolation and are incapable of blocking polar LiPSs, leading to active material loss and rapid capacity decay. In contrast, biomass-based materials, with their naturally abundant highly polar functional groups (such as –OH and –NH_2_) on the framework and tunable hierarchical pore networks, provide a natural material platform for the targeted interception of polysulfides. By utilizing polar networks and functional composite coating strategies, biomass-based separators achieve the “chemical anchoring” and “catalytic conversion” of polysulfides, thereby completely suppressing the shuttling effect and enhancing battery cycle life.

In terms of enhancing chemical affinity and anchoring effects, the intrinsic polar network of biomass macromolecules plays a key role in trapping. For example, Zhang et al. [121] constructed a highly interconnected three-dimensional aerogel separator using bacterial cellulose (BC). This separator forms chemical bonds with polysulfides through strong hydrogen bonding interactions, achieving efficient “capture” of LiPSs at the molecular scale. Simultaneously, its abundant three-dimensional nanochannels physically extend the diffusion paths of polysulfides, enabling dual blocking through both physical confinement and chemical anchoring.

In constructing a more potent synergistic interfacial barrier, the biomass membrane, combined with a catalytically active coating, achieves an integrated “interception–conversion” mechanism for polysulfides. For the more severe shuttling issues in high-sulfur-loading systems, relying solely on adsorption often leads to saturation of active sites, whereas introducing a catalytic conversion mechanism can completely sever the shuttling cycle. Wu et al. [122] composite two-dimensional Ti_3_C_2_T_X_ (MXene) and SnS_2_ nanoparticles onto a porous bacterial cellulose (PBC) substrate to construct a multifunctional composite membrane. This design combines the strong adsorption capacity of Ti_3_C_2_T_X_ as a Lewis acid with the electrocatalytic activity of SnS_2_, enabling the functional composite layer to firmly anchor free LiPSs while rapidly lowering their reaction activation energy, thereby catalyzing their rapid conversion into insoluble solid-phase Li_2_S_2_/Li_2_S.

Biomass-based separators have demonstrated effective chemical anchoring and catalytic conversion of lithium polysulfides. Nevertheless, several critical challenges remain. At high sulfur loadings and lean electrolyte conditions, the limited number of adsorption/catalytic sites often leads to saturation and eventual capacity fade. The long-term mechanical integrity of the separator under the large volume changes in the sulfur cathode is still inadequate. Moreover, most studies are conducted in coin cells; the performance of biomass separators in practical pouch cells with high sulfur loading and limited electrolyte remains largely unexplored.

#### 5.2.3. Solid-State and Quasi-Solid-State Batteries

All-solid-state and quasi-solid-state batteries (SSBs) are widely recognized as the ultimate form of next-generation high-energy-density energy storage due to their intrinsic safety, which completely eliminates the risk of thermal runaway associated with liquid organic electrolytes. However, traditional solid-state electrolyte systems have long been constrained by the “mechanical–kinetic interface” trade-off dilemma. Although solid-state polymer electrolytes (SPEs) possess excellent interfacial flexibility, they are limited by slow ion transport due to high crystallinity at room temperature, and their low-modulus matrix is highly susceptible to penetration by metal dendrites; conversely, inorganic ceramic electrolytes, while exhibiting superior mechanical strength, suffer from extremely high solid–solid contact resistance due to their inherent brittleness. To resolve this physical and chemical contradiction, biomass nanofibers—which possess ultra-high axial modulus and highly polar surfaces—were introduced into the matrix. Through a synergistic mechanism combining “multidimensional mechanical stress dissipation” and “polarity-induced decrystallization and interfacial fusion,” the mass transfer and interfacial barriers faced by high-energy-density solid-state batteries were completely overcome.

**Figure 17:**
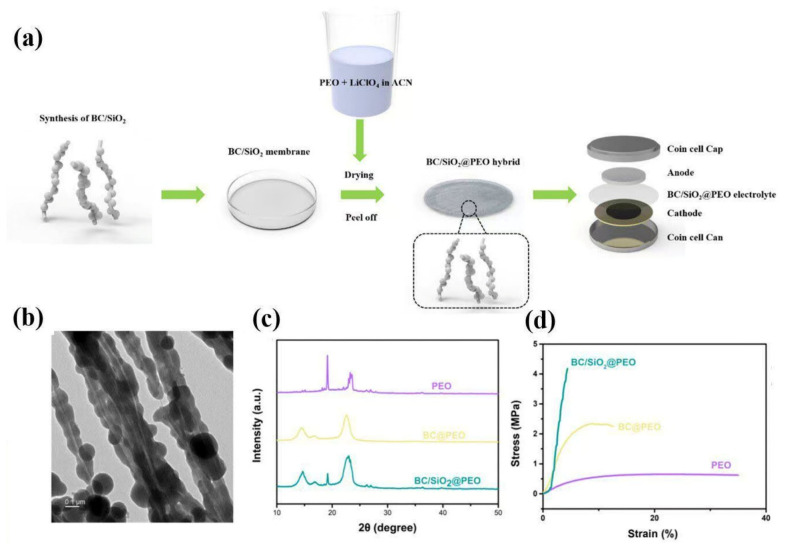
(**a**) Schematic diagram of the synthesis procedure for the BC/SiO_2_@PEO biomass gel-derived gel polymer electrolyte; (**b**) TEM image of BC/SiO_2_ nanofibers showing the conformal silica coating on the bacterial cellulose network; (**c**) XRD patterns of neat PEO, BC/SiO_2_, and BC/SiO_2_@PEO composites demonstrating a significant reduction in PEO crystallinity induced by the synergistic effect of BC hydroxyl groups and the SiO_2_ coating; (**d**) Stress–strain curves of PEO, BC@PEO, and BC/SiO_2_@PEO highlighting the dramatically enhanced mechanical robustness (nearly sevenfold increase in tensile strength) of the BC/SiO_2_@PEO composite. Adapted from Ref. [123] according to the Creative Commons Attribution—NonCommercial 3.0 Unported Licence.

In addressing the bottlenecks of mechanical fatigue and high crystallinity in polymer matrices, the three-dimensional network of plant cellulose demonstrates exceptional structural rigidity and a crystallization-suppressing effect. For example, as illustrated in Figure 17, Hong et al. [123] in situ coated the surface of bacterial cellulose nanofibers with SiO_2_ and used this as a scaffold to impregnate PEO polymer to construct a biomass gel-derived gel polymer electrolyte (GPE). In this design, the rigid BC-SiO_2_ interpenetrating gel network not only significantly enhanced the composite matrix’s resistance to mechanical deformation through a multidimensional mechanical stress dissipation mechanism but also physically and effectively blocked the longitudinal penetration of lithium dendrites; more crucially, the densely distributed polar hydroxyl groups (–OH) on the BC scaffold interact synergistically with the SiO_2_ coating, profoundly disrupting the ordered arrangement of PEO segments and inducing significant decrystallization. This dual mechanical and chemical intervention is reported to enhance the structural integrity of the electrolyte while promoting improved solid–solid interfacial contact and faster ion transport, enabling the full cell to exhibit excellent capacity retention during long-cycle cycling.

In addition to physical stress dissipation and decrystallization, the nitrogen-containing polar groups unique to chitin/chitosan derivatives provide a novel intervention pathway for deep chemical passivation at solid–solid interfaces. Zhou et al. [124] incorporated Mg^2+^-loaded modified carboxymethyl chitosan as a functional scaffold into a PVDF-HFP matrix to form a biomass gel-derived quasi-solid gel electrolyte. On the one hand, the abundant polar –OH and –NH_2_ groups on the chitosan segments, through a dense hydrogen-bonding network, not only enhance mechanical stress dissipation at the macroscopic level but also firmly anchor trace amounts of free solvent, thereby suppressing parasitic side reactions at the interface; On the other hand, this composite framework effectively drives the directed migration of Mg^2+^ toward the anode during electrochemical cycling, thereby in situ inducing the formation of a tough SEI layer rich in inorganic components (MgF_2_). This interface in situ reconstruction strategy, driven by the supramolecular gel notework, significantly broadens the electrochemical stability window of the electrolyte, enabling the high-voltage symmetric cell to maintain an ultra-low polarization voltage over an extremely long cycle life.

Although biomass nanofibers effectively reinforce polymer matrices and reduce crystallinity, solid-state and quasi-solid-state systems still face major challenges. The solid–solid interfacial impedance between the biomass-derived gel electrolyte and electrodes tends to increase during long-term cycling due to mechanical fatigue and volume changes. Achieving simultaneously high room-temperature ionic conductivity, excellent mechanical strength against lithium dendrite penetration, and long-term chemical stability remains difficult. In addition, the compatibility of biomass scaffolds with high-voltage cathodes and the development of solvent-free or green processing routes for large-area solid-state separators require further attention.

#### 5.2.4. Aqueous Zinc-Ion Batteries

Although aqueous zinc-ion batteries possess intrinsic advantages such as high safety, low cost, and environmental compatibility, their practical deployment remains severely hindered by persistent parasitic issues, including zinc (Zn) dendrite growth, the hydrogen evolution reaction (HER), Zn corrosion, and electrolyte leakage [125]. To address these challenges, biomass-derived hydrogel separators and gel electrolyte membranes have emerged as promising alternatives to conventional dry separators. Benefiting from their unique three-dimensional (3D) hydrated polymer networks, these gel systems can simultaneously facilitate in situ electrolyte retention, rapid ion transport, and intimate electrode/electrolyte interfacial contact, thereby mitigating these degradation mechanisms [56]. Biomass polymers, such as cellulose, bacterial cellulose, chitosan, and alginate, inherently feature abundant polar functional groups capable of forming stable and tunable gel networks via hydrogen bonding, ionic coordination, or covalent crosslinking. Concurrently, the strong electrostatic or coordination interactions between these polar moieties and Zn^2+^ can effectively regulate ion-transport behaviors.

Consequently, biomass polysaccharide-based hydrogel electrolytes have recently become a vital functional platform for suppressing Zn dendrite propagation, inhibiting HER, and stabilizing the electrode/electrolyte interface. For instance, Tong et al. [126] constructed a dual-crosslinked cellulose-based hydrogel electrolyte, which delivered a high ionic conductivity of 18.46 ± 0.39 mS cm^−1^ and a wide electrochemical stability window of 0–2.23 V. As a result, the Zn||Zn symmetric cell operated stably for over 3000 h at 1 mA cm^−2^, effectively suppressing Zn dendrite growth. Chitosan-based gel systems have also shown favorable ion-transport and interfacial regulation capabilities. Garcia-Castrillo et al. [70] developed a hybrid gel electrolyte by integrating chitosan with Zn-MOF-74, enabling a Zn||α-MnO_2_ full cell to deliver a discharge capacity of 106 mAh g^−1^ at 1.0 A g^−1^, representing an approximately 71% improvement compared with a glass-fiber separator. In addition, Ma et al. [57] prepared a Ca^2+^/Zn^2+^ dual-ion-crosslinked alginate hydrogel electrolyte, in which the “egg-box” conformation network and secondary Zn^2+^ crosslinking synergistically regulated water activity and ion confinement. The corresponding Zn||MnO_2_ full cell retained 62.03% of its capacity after 700 cycles. Collectively, these advancements underscore that the structural engineering of biomass polysaccharides, through dual-crosslinking, ionic coordination, and hybrid network design, can profoundly optimize ion transport, enhance interfacial stability, and ensure uniform Zn deposition, validating their vast potential for next-generation AZIBs.

Biomass-derived hydrogel and gel electrolyte membranes have significantly mitigated zinc dendrite growth and improved interfacial contact. However, persistent challenges include water-induced side reactions (hydrogen evolution and zinc corrosion), especially under lean electrolyte or high current density conditions. The long-term stability of the gel network against dehydration, swelling, and mechanical puncture by zinc dendrites in practical pouch cells is still insufficient. Moreover, the performance of biomass gel separators in seawater-based or hybrid electrolytes, as well as their compatibility with high-mass-loading cathodes, needs systematic investigation for real-world applications.

### 5.3. Summary of the Performance Evaluation System and Quantitative Analysis of Data

In summary, biomass-based composite separators have demonstrated high versatility and superior mechanisms across applications ranging from traditional lithium-ion batteries to various emerging energy storage systems. To more intuitively establish the technical standing of functionalized biomass-based separators at current battery interfaces, this section provides a systematic quantitative analysis and cross-comparison of core performance evaluation parameters (covering thermodynamic, kinetic, and electrochemical long-cycle metrics) from recent representative studies (see Table 6).

The following clear conclusions can be drawn from the data trends in the multidimensional evaluation system: First, in the thermal/mechanical safety dimension, nearly all cross-linked, hybridized, or structurally engineered biomass-based separators have completely surpassed the physical limit of approximately 130 °C for melt shrinkage in commercial polyolefin separators (PE/PP). They generally achieve a thermal shrinkage rate approaching 0% at extremely high temperatures of 150 °C or even 200 °C, while tensile strength is significantly enhanced. Second, in terms of interfacial mass transfer kinetics, thanks to a rich polar network and optimized topological pore channels, the electrolyte absorption rate of biomass-based separators typically reaches 2 to 5 times that of commercial separators (generally >200%). This not only enables room-temperature ionic conductivity to generally exceed the 1. 0 mS cm^−1^ threshold, but the synergy between oxygen- and nitrogen-containing polar groups and inorganic fillers also significantly increases the number of cations (such as Li^+^/Na^+^) transported to the 0.6–0.8 range, fundamentally mitigating concentration polarization at high rates of discharge. Finally, regarding battery service life, whether suppressing dendrites in high-areal-capacity lithium metal symmetric cells, trapping polysulfides in lithium–sulfur batteries, or reducing interfacial impedance in solid-state batteries, complete cells equipped with customized biomass-based separators demonstrate exponentially improved cycle life and excellent capacity retention.

Overall, the quantitative data presented above fully confirms that rational functionalization design has elevated biomass materials from a low-cost “substitute substrate” to a “multifunctional platform” capable of comprehensively reshaping the mechanical, thermal, and kinetic boundaries of battery interfaces.

## 6. Conclusions and Outlook: Challenges and Future Trends

### 6.1. Existing Industrialization Bottleneck and Conventional Optimization Routes

This review has systematically examined biomass-based battery separators derived from cellulose, chitin/chitosan, and lignin, spanning molecular characteristics, gelation and film-forming processes (electrospinning, solution casting/NIPS, nonwoven technology, and hydrogel-assisted routes), advanced functionalization strategies (surface chemical modification, multicomponent hybridization into metallogels, and refined three-dimensional gel-network engineering), and cross-system performance validation in lithium-ion, lithium–sulfur, sodium/potassium-ion, aqueous zinc-ion, and solid-state batteries. These biomass-derived hydrogels, dried gels, colloidal gels, and supramolecular networks not only inherit intrinsic advantages in thermal stability (>200–300 °C dimensional integrity) and electrolyte affinity (contact angles often < 30° and liquid uptake > 200%), but also function as versatile platforms for constructing non-gel functional separators that actively suppress metal dendrite growth via supramolecular interactions, immobilize soluble polysulfides, and lower solid–solid interfacial impedance.

Despite impressive laboratory progress, several fundamental and interrelated challenges continue to prevent biomass-derived gel separators from transitioning into commercially viable products. A more critical appraisal of these bottlenecks is essential for guiding the next phase of research.

Challenge 1: The persistent mechanical-strength versus ionic-conductivity trade-off Strategies that successfully raise tensile strength and puncture resistance (dense chemical crosslinking, elevated crystallinity, or high loadings of inorganic fillers) almost invariably compress the gel-network porosity and tortuosity, reducing room-temperature ionic conductivity. Typical pure cellulose nonwoven membranes achieve 29–49 MPa tensile strength but only 0.7–1.0 mS cm^−1^ conductivity; further reinforcement to >50 MPa often drops conductivity below 0.5 mS cm^−1^. Conversely, highly porous electrospun or freeze-dried gels deliver >2 mS cm^−1^ but suffer tensile strengths of only 1–15 MPa, insufficient for high-speed winding or metal-anode volume expansion. This trade-off is intrinsic to current one-dimensional fiber or isotropic hydrogel architectures. Future work must deliberately engineer multi-scale hierarchical gel networks (nanofibril skeletons + mesoporous secondary networks + gradient densification) that decouple load-bearing and ion-transport pathways.

Challenge 2: Structural and compositional inconsistency of natural feedstocks. Unlike petrochemical polyolefins with tightly controlled molecular-weight distributions, biomass sources exhibit substantial seasonal, regional, and species-dependent variability in fiber diameter, crystallinity index (CrI 40–92%), residual hemicellulose/lignin/pectin content, and surface charge density. These variations translate directly into non-reproducible pore-size distributions, thickness tolerances, and electrochemical performance, rendering batch-to-batch quality control extremely difficult for industrial production. Without standardized, battery-grade purification protocols (e.g., residual ash < 50 ppm, heavy-metal limits, and zeta-potential windows) and rapid in-line characterization methods, large-scale manufacturing remains impractical.

Challenge 3: Limited compatibility with existing industrial manufacturing infrastructure. Current laboratory routes—especially high-voltage electrospinning and freeze-drying—are energy-intensive, low-throughput, and poorly matched to the continuous roll-to-roll biaxial-stretching lines optimized for PP/PE separators. Wet-laid nonwoven and aqueous solution-casting processes are more scalable and greener, yet still require significant capital investment for solvent recovery, wastewater treatment, and precise thickness control under high-speed conditions. A realistic near-term pathway is the hybridization of mature papermaking/nonwoven technology with minimal green chemical modification, followed by continuous hot-pressing or UV-crosslinking stations that can be retrofitted onto existing lines.

Challenge 4: Absence of standardized evaluation protocols tailored to biomass- and gel-based separators. Existing standards, such as IEC 61960-3 (Geneva, Switzerland, 2017) for secondary lithium cells and batteries for portable applications and ASTM D882 (PA, USA, 2026.) for tensile testing of thin plastic sheeting, mainly address general battery performance or conventional polymer-film mechanical testing rather than the specific failure modes of biomass- and gel-based separators [127,128]. These failure modes include wet puncture strength under electrolyte-swollen conditions, long-term dimensional stability under lean-electrolyte operation, gel-network aging and chain scission in the presence of transition-metal ions or HF, high-humidity storage effects, and realistic thermal-runaway propagation behavior in multilayer pouch cells. Without community-agreed protocols and round-robin inter-laboratory validation, claimed performance advantages remain difficult to compare, and industrial risk assessment remains incomplete.

Challenge 5: Incomplete life-cycle and techno-economic assessment. Although biomass feedstocks are renewable, the energy and chemical inputs of purification, TEMPO oxidation, electrospinning solvents, and freeze-drying can erase the environmental benefit if not carefully optimized. Rigorous cradle-to-grave LCA studies that quantify greenhouse-gas emissions, water footprint, and end-of-life recyclability relative to ceramic-coated polyolefin separators are still scarce. Parallel techno-economic analyses that include realistic yield, capital expenditure, and scale-up factors are equally needed to identify the true cost-competitiveness window.

Addressing these five interconnected bottlenecks requires a shift from isolated materials optimization toward integrated process–structure–performance–sustainability design. Priority research directions include: (i) hierarchical multi-scale gel architectures that simultaneously satisfy mechanical and transport requirements; (ii) closed-loop green purification and continuous solvent-free manufacturing platforms; (iii) open, standardized testing protocols and shared databases for biomass separators; and (iv) early-stage LCA-guided materials selection. Progress on these fronts will determine whether biomass gel separators remain laboratory curiosities or become genuine industrial alternatives for high-safety, high-energy-density batteries.

### 6.2. Data-Driven Design via Artificial Intelligence and Materials Informatics

Driven by the rapid integration of artificial intelligence (AI) and materials informatics into electrochemical energy storage research, data-driven research paradigms have broken the limitations of traditional trial-and-error experimental workflows and provided innovative routes for the rational design and targeted optimization of next-generation biomass gel separators. The core research directions cover machine learning-assisted molecular design, data-based electrochemical performance prediction and automated high-throughput material screening.

For AI-aided structural engineering of biomass membranes, machine learning models can establish quantitative mapping relationships between biomass molecular skeletons, grafted polar functional groups, inorganic filler loading ratios and separator thermo-electrochemical performance based on massive experimental datasets. This framework avoids redundant repeated synthesis and characterization experiments, and guides targeted surface functionalization strategies (e.g., TEMPO oxidation, silane grafting) for cellulose, chitosan and lignin substrates. Generative artificial intelligence further enables biomimetic design of multi-scale porous gel networks that simultaneously balance high porosity, suppressed thermal shrinkage and enhanced mechanical modulus, optimizing ion transport pathways from the molecular topological perspective.

Machine learning-based performance prediction represents the most mature application scenario at present. By aggregating literature data covering thermal decomposition temperature, electrolyte absorption, ionic conductivity, tensile strength and long-cycle retention of biomass separators, gradient boosting and neural network prediction architectures can be constructed. Input variables including biomass feedstock type, modification scheme and fabrication parameters allow rapid forecasting of comprehensive membrane performance, drastically cutting the number of experimental groups required for multivariate optimization. Multi-objective optimization algorithms can also be deployed to reconcile conflicting performance indicators such as mechanical robustness and ionic permeability, thermal stability and electrolyte retention.

High-throughput screening technology combines automated film-forming equipment, in situ microscopic and electrochemical characterization with AI data analysis, enabling batch testing of hundreds of biomass raw materials and composite formulations. This efficiently resolves the cumbersome screening challenges arising from diversified biomass sources and numerous gel modification pathways.

Nevertheless, this research field still suffers from obvious constraints, including non-uniform experimental testing standards and incomplete standardized descriptor systems for biomass gel network structures. Future work should focus on constructing unified open databases dedicated to cellulose/chitin/lignin separators, coupled with multi-physics finite element simulation and large language model auxiliary analysis, to accelerate the iterative development of industrializable high-performance biomass-based porous membranes.

## Figures and Tables

**Table 2:** Comparison of Major Sources of Chitin/Chitosan.

Source Type	Representative Raw Materials	Content Range	Key Advantages	Limitations
Crustaceans	Shrimp shells, crab shells	17.50–23.75% [16]	Abundant raw materials, mature industry	Requires decalcification and deproteinization
Insects	Beetles, locusts, mealworms	1.2–60.0% [14]	Rapid growth, low land requirements, environmentally friendly	High variability in content
Fungi	Fermentation residues, edible mushrooms	0.5–40.0% [19]	High purity, free of heavy metals and allergens	Extraction process is somewhat complex
Mollusks	Squid beaks, cuttlefish	Typically 4–10% in shells; up to 20–40% in some pens [21]	High degree of polymerization, good mechanical properties	Limited yield

**Table 3:** Comparison of Intrinsic Physicochemical Properties Between Major Biomass Fibers and Polyolefin Separators.

Property	Cellulose	Chitin/Chitosan	Lignin	Polyolefin (PP/PE)	Major Factors Affecting Membrane Performance
Molecular structure	Linear β–1,4–glucose chains, Degree of polymerization (DP) 800–10,000 [24]	β–1,4–glucosamine chains, Deacetylation degree 80–95% [1]	3D aromatic cross-linked network, predominantly β–O–4	Linear/branched hydrocarbon chains	Determines pore network and ion channel type
Crystallinity	Crystallinity Index (CrI) 40–70% [25]	CrI 59–92% [26]	Amorphous	Semi-crystalline (40–60%)	High crystallinity enhances thermal/mechanical stability; low crystallinity promotes pore formation
Major functional groups	–OH (3 per unit)	–OH & –NH_2_	Phenol/ester –OH, –OCH_3_, –SO_3_H (after sulfonation)	None (hydrophobic)	Controls hydrophilicity, ion selectivity, and interfacial stability
Thermal stability	Decomposition onset 300–350 °C [27]	Decomposition onset 259–350 °C [28]	Decomposition onset 200–400 °C [1]	Melting point 135–165 °C [2]	Significantly increases the battery safety threshold (suppresses thermal runaway)
Hydrophilicity	Contact angle < 30° Liquid absorption > 200% [29]	Contact angle < 40° Liquid absorption > 200% [30]	Contact angle 18.3–40°, liquid absorption > 200% [1]	Contact angle > 118.4°, liquid absorption rate < 200% [31]	Accelerates electrolyte wetting and improves rate capability
Mechanical properties	Tensile strength 20–80 MPa [1]	Tensile strength 47–120 MPa [1]	Tensile strength < 4 MPa (Increased after fiberization) [32]	Tensile strength > 100 MPa [33]	Support assembly and bending require composite reinforcement to mitigate brittleness
Porosity (typical)	>50% [1]	>50% [6]	>50% [33]	40–50% [34]	Effects on ionic conductivity and dendrite inhibition

**Table 5:** Comparison evaluation of major fabrication routes for biomass-based battery separators.

Fabrication Route	Structural Control	Mechanical Properties	Scalability & Manufacturing Practicality	Best Suited Biomass Materials	Preferred Applications/Rationale	Main Limitations
Electrospinning	Excellent (nanoscale fibers, tunable pore size & high surface area)	Generally low (requires reinforcement)	Low (batch process, high energy consumption)	Chitosan, lignin blends, cellulose acetate	High ion selectivity, nanofibrous gel networks, high-rate or solid-state batteries	Low mechanical strength, poor scalability, solvent use
Solution Casting (NIPS)	Good (controllable sponge-like or finger-like pores)	Moderate	Medium (continuous possible with solvent recovery)	Regenerated cellulose, chitosan	Precise thickness control, good uniformity, versatile for lab-to-pilot scale	Often requires organic solvents, moderate mechanical strength
Nonwoven Technology (esp. wet-laid)	Moderate (micro- to submicron pores)	High (typically 29–49 MPa)	High (compatible with industrial papermaking, solvent-free)	Plant cellulose, bacterial cellulose	Large-scale production, high mechanical robustness, low cost, green processing	Limited nanoscale precision, less suitable for rigid materials like pure lignin
Hydrogel-assisted Film Formation	Good (preserves native 3D network)	High after post-treatment (hot-pressing/ crosslinking)	Low to Medium	Bacterial cellulose, alginate, chitosan gels	Preservation of native gel topology, high porosity & electrolyte retention, solid-state or gel electrolytes	Difficult to scale continuously, requires additional strengthening steps

**Table 6:** Comparison of performance among different biomass-based separators.

Separator Type/ Biomass Source/ Modification Strategy	Battery System	Ionic Conductivity (mS cm^−1^)	Thermal Shrinkage (% @150 °C)	Liquid Absorption (%)	Ion Transference Number	Cycle Performance (Capacity Retention/ Cycle Count/Rate)	References
Commercial PP/PE	LIBs	0.5–1.0	40–80	150–250	0.3–0.4	70–80%/ 200–300/1 C	Commercial standard
Cellulose/PI- COOH (hydrogen-bond crosslinked)	LIBs (High Safety)	0.51	~0	638	0.51	90%/200/1 C	[103]
Lignin/PI (π–π stacking + hydrogen bonding blend)	LIBs (High Safety)	1.78	~0	592	0.787	95.1%/100/1 C	[105]
Bacterial Cellulose (Gradient Pore Design)	Li-S	2.15	~0	440	0.71	83%/200/0.5 C	[110]
Ti_3_C_2_T_X_-SnS_2_- PBC (layer-by-layer self-assembly + sputtering)	Li-S	2.171 (180 °C)	~0	385	—	71.2% increase/ 500/ 0.2 C	[111]
BC/SiO_2_@PEO (GPE)	All-solid- state/ Semi-solid	Significant improvement	0 (150 °C)	—	—	94%/200/1 C	[115]
CHI@Mg-PVDF-HFP (GPE)	All-solid- state/ Semi-solid	1.93 × 10^−4^ (30 °C)	0	—	0.62	86.6%/1000/2 C	[76]

## Data Availability

No new data were created in this study. All data discussed in this review were obtained from previously published literature and are available from the cited sources.
